# Ionic Liquids Impact the Bioenergy Feedstock-Degrading Microbiome and Transcription of Enzymes Relevant to Polysaccharide Hydrolysis

**DOI:** 10.1128/mSystems.00120-16

**Published:** 2016-12-13

**Authors:** Yu-Wei Wu, Brendan Higgins, Chaowei Yu, Amitha P. Reddy, Shannon Ceballos, Lawrence D. Joh, Blake A. Simmons, Steven W. Singer, Jean S. VanderGheynst

**Affiliations:** a Joint BioEnergy Institute, Emeryville, California, USA; b Graduate Institute of Biomedical Informatics, College of Medical Science and Technology, Taipei Medical University, Taipei, Taiwan; c Biological and Agricultural Engineering, University of California, Davis, Davis, California, USA; d Biosystems Engineering, Auburn University, Auburn, Alabama, USA; e Biological and Materials Science Center, Sandia National Laboratories, Livermore, California, USA; f Biological Systems and Engineering Division, Lawrence Berkeley National Laboratory, Berkeley, California, USA; Oregon State University

**Keywords:** 1-ethyl-3-methylimidazolium acetate, cellulase, hemicellulase, ionic liquid

## Abstract

Ionic liquid (IL) pretreatment is a promising approach for the conversion of lignocellulose to biofuels. The toxicity of residual IL, however, negatively impacts the performance of industrial enzymes and microorganisms in hydrolysis and fermentation. In this study, a thermophilic microbial community was cultured on switchgrass amended with various levels of the ionic liquid 1-ethyl-3-methylimidazolium acetate. Changes in the microbial community composition and transcription of genes relevant to IL tolerance and lignocellulose hydrolysis were quantified. Increasing the level of IL to 0.1% (wt) led to increased levels of relative abundance and transcription in organisms of the phylum *Firmicutes*. Interestingly, IL concentrations of up to 1% (wt) also resulted in greater xylanase transcription and enzyme activity as well as increased transcription of endoglucanase, beta-glucosidase, and IL tolerance genes compared to communities without IL. IL levels above 1% (wt) resulted in decreased enzyme activity and transcription of genes involved in lignocellulose hydrolysis. The results indicate that moderate levels of IL select for thermophilic microorganisms that not only tolerate IL but also effectively hydrolyze lignocellulose from switchgrass. Discovery of IL-tolerant organisms and enzymes is critical for the development of biological processes that convert IL-pretreated biomass to biofuels and chemicals. Employing metatranscriptomic analysis of enrichment cultures can facilitate the discovery of microorganisms and enzymes that may be active in the presence of toxic compounds such as ionic liquids.

**IMPORTANCE** Pretreatment using ionic liquids (IL) is a promising approach for the conversion of lignocellulose to biofuels. Because IL can be inhibitory to enzymes and microorganisms involved in downstream hydrolysis and fermentation steps, discovery of IL-tolerant organisms and enzymes is critical for advancing this technology. Employing metatranscriptomics in the analysis of IL-enriched cultures facilitated tracking of dynamic changes in a complex microbial community at the level of gene transcription and doing so with genome resolution. Specific organisms were discovered that could simultaneously tolerate a moderate IL concentration and transcribe a diverse array of cellulolytic enzymes. Gene sequences of cellulolytic enzymes and efflux pumps from those same organisms were also identified, providing important resources for future research on engineering IL-tolerant organisms and enzymes.

## Abstract (precis)

Pretreatment using ionic liquids (IL) is a promising approach for the conversion of lignocellulose to biofuels. Because IL can be inhibitory to enzymes and microorganisms involved in downstream hydrolysis and fermentation steps, discovery of IL-tolerant organisms and enzymes is critical for advancing this technology. Employing metatranscriptomics in the analysis of IL-enriched cultures facilitated tracking of dynamic changes in a complex microbial community at the level of gene transcription and doing so with genome resolution. Specific organisms were discovered that could simultaneously tolerate a moderate IL concentration and transcribe a diverse array of cellulolytic enzymes. Gene sequences of cellulolytic enzymes and efflux pumps from those same organisms were also identified, providing important resources for future research on engineering IL-tolerant organisms and enzymes.

## INTRODUCTION

The production of biofuels from plant biomass offers opportunities for improved environmental sustainability and energy security. Lignocellulosic sources of biomass offer great promise in terms of meeting demands for low-carbon liquid fuels with limited impact on land use (1–3). One significant challenge associated with production of biofuels from lignocellulose is its recalcitrance with respect to bioconversion (4), and major research investments have been aimed at developing economical methods to improve crops and reduce recalcitrance for conversion to fuels (5, 6). Pretreatment with ionic liquids (ILs) is one promising deconstruction method; however, residual IL from pretreatment is inhibitory to downstream biological conversion processes and extensive washing (~100 liters water kg^−1^ dry biomass) is required to decrease IL concentrations to levels that can be tolerated by commercial enzymes and microorganisms (7, 8). The level of required biomass washing could be significantly reduced if enzymes and organisms associated with downstream processes were more IL tolerant. Several solutions to this challenge include identifying cellulases and xylanases that remain active in the presence of ILs and genetic modification of industrial strains of microorganisms to improve IL tolerance (9–11).

In prior work, it was demonstrated that organisms tolerant of IL were present in thermophilic cultures enriched with high levels of solids (12, 13), suggesting that they may be a source of industrially relevant enzymes and microorganisms. However, those works either focused on mechanisms of IL tolerance in individual organisms or analyzed microbial community response to IL treatment more generally through 16S rRNA gene pyrosequencing. Employing metagenomic and metatranscriptomic tools could facilitate discovery of potentially IL-tolerant lignocellulolytic enzymes within complex microbial communities and could facilitate mapping of those enzymes to specific organisms.

The first objective of the present work was to identify organisms within a thermophilic, switchgrass-adapted community that increase in relative abundance and transcription activity in response to an increased IL concentration. The second objective was to examine differential transcription levels of genes involved in IL tolerance and polysaccharide hydrolysis as IL concentrations increased. The identified organisms and genes could be a resource for thermophilic bioconversion systems that employ IL pretreatment.

## RESULTS

### Influence of IL amendment on microbial activity and extracted enzyme activity.

Switchgrass samples amended with [C_2_mim][OAc] (1-ethyl-3-methylimidazolium acetate) were examined in this study at four levels: 0%, 0.5%, 1%, and 2% IL. The cumulative carbon dioxide evolution rate (cCER) decreased as the IL concentration increased (Table 1) (14). The cCER seen with switchgrass amended with 2% IL was significantly lower than the cCER from samples amended with 0% and 0.5% IL. There was no significant effect of IL amendment on feedstock reduction.

**TABLE 1:** Final (day 7) cCER and feedstock reduction of incubations with switchgrass treated with different levels of [C_2_mim][OAc]^a^

Feedstock	Mean cCER (mg CO_2_-g^−1^ dry matter)	% mean reduction (wt)
0% IL	299 (11) A	6.0 (2.0) A
0.5% IL	296 (24) A	8.2 (1.7) A
1% IL	268 (7) A,B	9.3 (2.3) A
2% IL	241 (16) B	7.2 (2.0) A

a Standard deviations are given in parentheses. *n* = 3. Means followed by the same letter are not statistically different at α = 0.05. cCER, cumulative carbon dioxide evolution rate (cCER).

**FIG 1:**
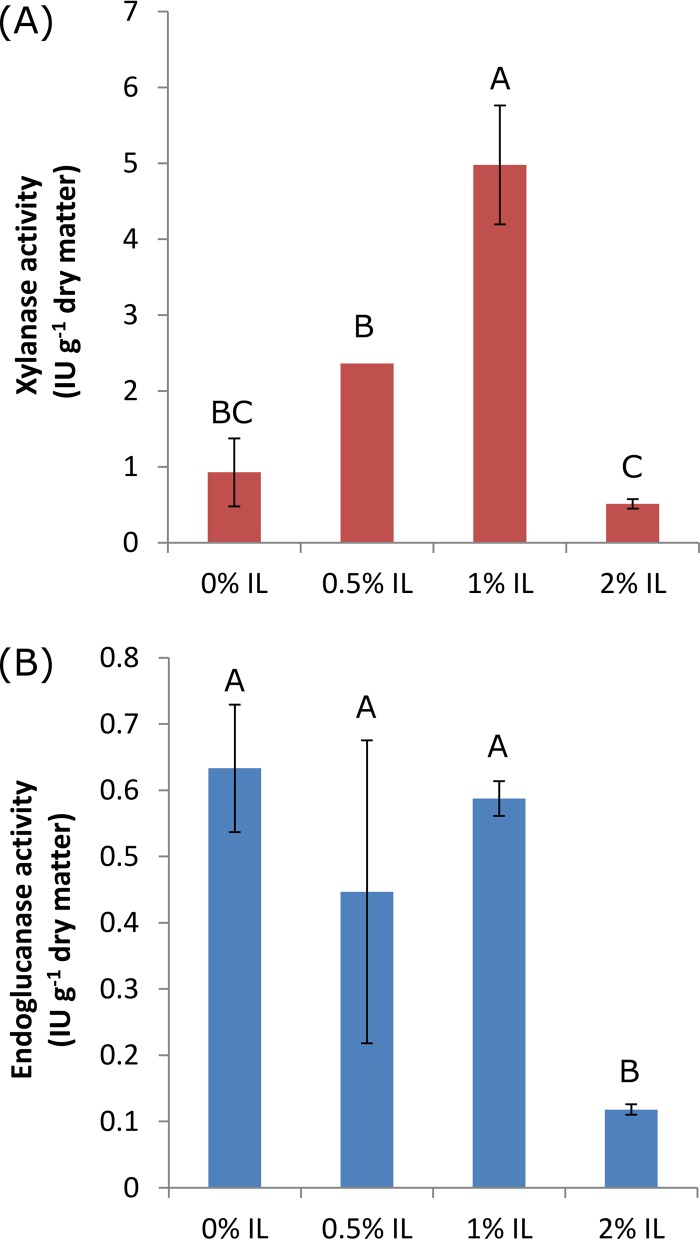
Activity of enzymes extracted from compost-derived microbial communities incubated on switchgrass amended with different levels of the ionic liquid ([C_2_mim][OAc]). (A) Mean xylanase activities. An outlier for xylanase activity associated with the 0.5% IL-adapted community was observed and removed, resulting in no standard deviation calculated. (B) Mean endoglucanase activity levels. Data in columns labeled with the same letter(s) are not statistically different at α = 0.05.

Enzyme activities at the end of 7-day incubation periods for each treatment are shown in Fig. 1. The xylanase activities increased as the IL concentration increased from 0% to 1% but decreased at the 2% IL concentration. The endoglucanase activity levels were between 0.45 and 0.62 IU·g^−1^ dry matter for switchgrass amended with 0% to 1% IL and were significantly higher than the level observed in switchgrass amended with 2% IL (Fig. 1B).

### Influence of IL amendment on microbial community composition.

**FIG 2:**
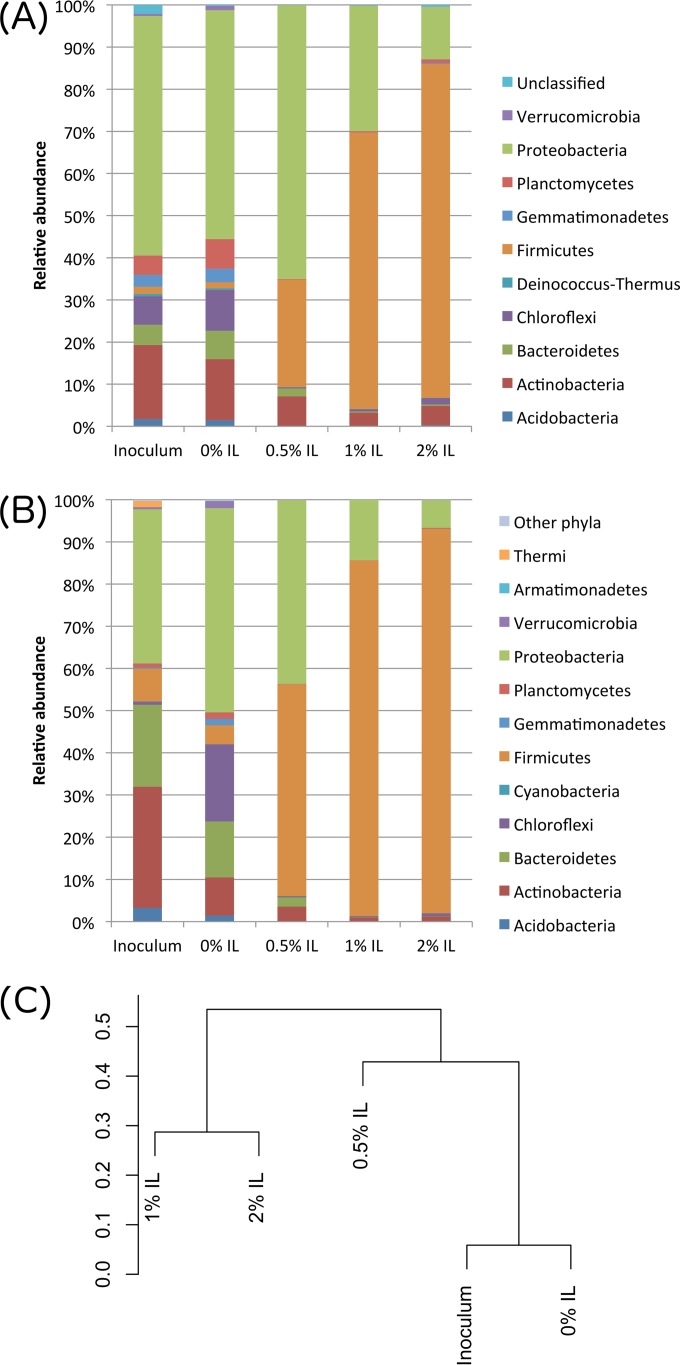
Relative abundances of phyla in the microbial communities under conditions of various IL concentrations. (A and B) DNA sequence data were binned and assigned to the nearest taxonomic classification based on full metagenome analysis (A) and iTag 16S ribosomal RNA analysis (B). (C) Distance tree showing separation of metagenomes.

Metagenomes of the initial inoculum and four microbial communities adapted to various IL concentrations were sequenced using an Illumina HiSeq 2000 system. The sizes of the metagenomes are summarized in Table S1 in the supplemental material. Metagenomic reads were then put together and coassembled, yielding 979,021 scaffolds, among which there were 140,763 scaffolds that were al least 1,000 bp in size. The maximum and N50 lengths of the scaffolds were 531,429 and 3,418 bp, respectively. Binning of the coassembled metagenomic scaffolds using MaxBin 2.0 (15) successfully recovered 138 individual genomes (Table S2). Levels of coverage of the genomes were also estimated for the five samples by MaxBin (Table S3), as were the mapping rates of sequencing reads (Table S4). After predicting the phyla of these recovered genomes, we found that the relative abundance of *Firmicutes* increased with the ionic liquid concentration (Fig. 2A). This result was consistent with the 16S rRNA gene analysis data (Fig. 2B).

10.1128/mSystems.00120-16.2 Table S1  Data set sizes of the 16S amplicons, metagenomes, and metatranscriptomes. Download Table S1, PDF file, 0.02 MB. Copyright © 2016 Wu et al. 2016 Wu et al. This content is distributed under the terms of the Creative Commons Attribution 4.0 International license.

10.1128/mSystems.00120-16.3 Table S2  Taxonomic and genome information of the recovered genome bins. Download Table S2, PDF file, 0.3 MB. Copyright © 2016 Wu et al. 2016 Wu et al. This content is distributed under the terms of the Creative Commons Attribution 4.0 International license.

10.1128/mSystems.00120-16.4 Table S3  The coverages and relative abundance ratios estimated for the genomes recovered from the metagenomes. Download Table S3, PDF file, 0.3 MB. Copyright © 2016 Wu et al. 2016 Wu et al. This content is distributed under the terms of the Creative Commons Attribution 4.0 International license.

10.1128/mSystems.00120-16.5 Table S4  Mapping rates of genomic and transcriptomic sequencing reads against the coassembled metagenome. Download Table S4, PDF file, 0.04 MB. Copyright © 2016 Wu et al. 2016 Wu et al. This content is distributed under the terms of the Creative Commons Attribution 4.0 International license.

In communities with no IL (inoculum and 0% IL), members of the phylum *Proteobacteria* predominated. The no-IL communities generally had higher diversity, richness, and evenness than the high-IL communities (Table S5). Comparing the 0% and 2% IL cases, Shannon diversity values were 2.97 and 1.74, richness values were 190 and 143, and Pielou’s evenness values were 0.57 and 0.35, respectively. This suggests that only specific organisms could tolerate the high-IL environment, leading to a decrease in the relative abundances of many species. The transition of the dominant phylum from *Proteobacteria* to *Firmicutes* with increasing IL content suggests that *Firmicutes* organisms were better able to resist the toxicity of ionic liquid.

10.1128/mSystems.00120-16.6 Table S5  Shannon diversity and richness and Pielou’s evenness values for microbial communities under conditions of different treatments. Download Table S5, PDF file, 0.04 MB. Copyright © 2016 Wu et al. 2016 Wu et al. This content is distributed under the terms of the Creative Commons Attribution 4.0 International license.

A distance tree based on the abundances of the recovered genomes also clearly indicated that ionic liquid had a large impact on the species composition of the microbial population (Fig. 2C). Samples without IL (inoculum and 0% IL) were clustered together, as were samples with high IL content (1% and 2% IL). The 0.5% IL sample, which was the sample in which the transition from *Proteobacteria* to *Firmicutes* was most apparent, is located at a branch between the two groups of samples.

### Analysis of gene transcription levels in the recovered genomes.

**FIG 3:**
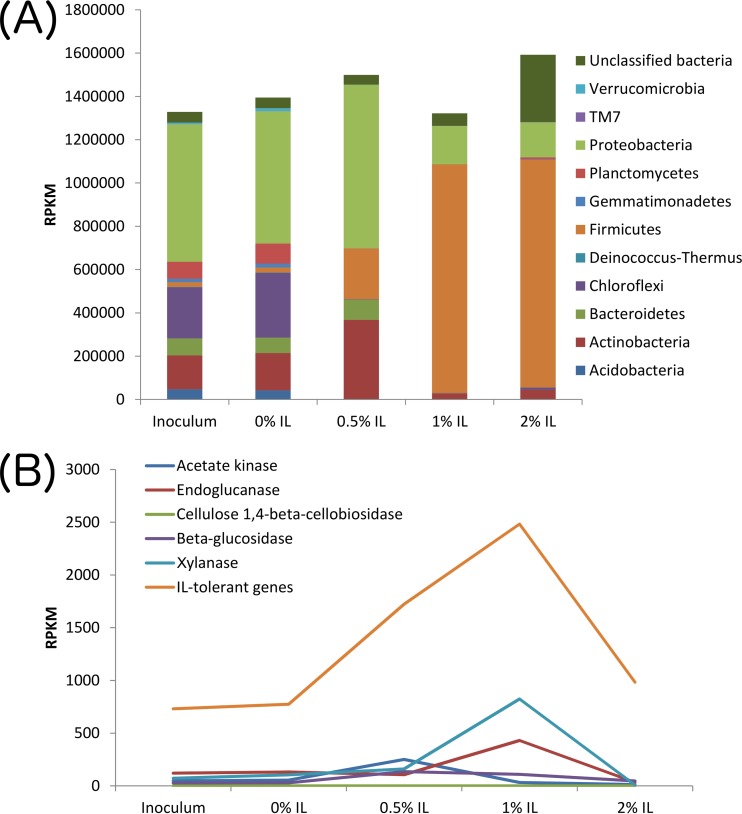
Total normalized transcript read values (RPKM) under conditions of various IL concentrations based on mRNA sequencing. (A) Transcript reads disaggregated based on phylum. (B) Total transcript reads based on gene function.

To quantify gene transcription, the metatranscriptomic sequencing reads were mapped to the genes predicted from the coassembled metagenomes to obtain the normalized read counts (reads per kilobase per million reads [RPKM]). The mapping rates of the metatranscriptomic reads from the five samples are also shown in Table S4. The genome expression levels were calculated by summing the RPKM values of genes that belong to each genome. Genes predicted from unbinned scaffolds were also assigned to the phylum-level taxonomy using MEGAN (see Materials and Methods). We observed that abundant bacterial phyla exhibited the greatest total gene transcription: *Proteobacteria* in the inoculum and 0% IL samples as well as *Firmicutes* in the 1% and 2% IL samples (Fig. 3A). Moreover, only a small number of genomes were responsible for the majority of transcription under high-IL conditions (a summary of the RPKM values for each genome bin can be found in Table S6). For instance, the top 5 genomes accounted for 56% and 65% of prokaryotic gene transcription at 1% and 2% IL, respectively, whereas the top 5 genomes in the inoculum and 0% IL samples accounted for only 46% and 42% of the prokaryotic gene transcription. This further suggests that IL selects for particular resilient organisms.

10.1128/mSystems.00120-16.7 Table S6  Summarized RPKM values for the genome bins. Download Table S6, PDF file, 0.1 MB. Copyright © 2016 Wu et al. 2016 Wu et al. This content is distributed under the terms of the Creative Commons Attribution 4.0 International license.

In the 0.5% IL sample, the greatest total gene transcription was observed in *Firmicutes* and *Proteobacteria*. Since *Proteobacteria* were not particularly abundant in the 1% and 2% IL samples whereas *Firmicutes* were rare in the inoculum and 0% IL samples, the existence of both phyla in the 0.5% IL sample as the two most abundant populations further suggests that 0.5% IL served as a transition point for the microbial community.

We were also interested in the effect of the IL concentration on transcription of genes relevant to biomass hydrolysis. We therefore used metatranscriptomics to identify organisms with genes related to cellulose and hemicellulose degradation, including genes encoding endoglucanases, cellulose 1,4-beta-cellobiosidases, beta-glucosidases, and xylanases (Fig. 3B). We also examined the transcription of genes that may confer tolerance of ionic liquid or similar quaternary ammonium cations (IL tolerance genes) and analyzed acetate kinase transcription to understand if organisms were utilizing acetate obtained from [C_2_mim][OAc] as a carbon source. The 1% IL community had the highest transcription of endoglucanase, xylanase, and IL tolerance genes, while the beta-glucosidase genes were most highly transcribed in the 0.5% IL sample. Acetate kinases were also most highly expressed in the 0.5% IL sample, suggesting that certain organisms may be able to utilize acetate as a carbon source at moderate IL levels. The transcription levels of cellulose 1,4-beta-cellobiosidase were very low in all samples, indicating that the microbial communities may not utilize this enzyme extensively for cellulose degradation.

**FIG 4:**
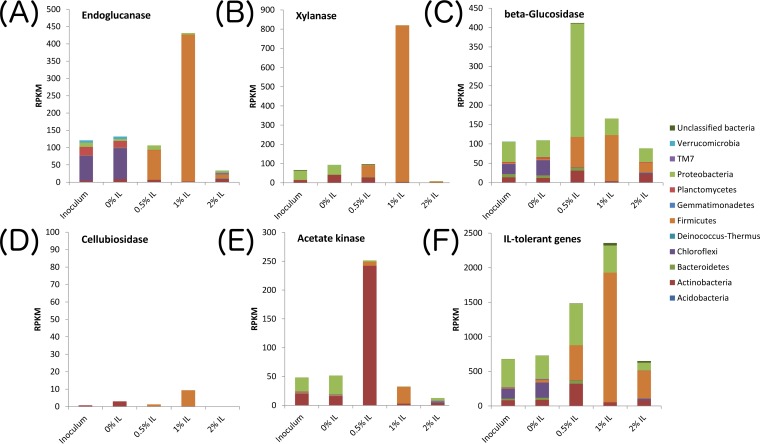
Transcription levels for genes of interest under conditions of different IL concentrations, disaggregated by phyla. (A) Endoglucanase genes. (B) Xylanase genes. (C) Beta-glucosidase genes. (D) Cellulose 1,4-beta-cellobiosidase genes. (E) Acetate kinase genes. (F) Ionic liquid tolerance genes.

The transcripts of these genes were also mapped to the metagenomic bins based on phyla (Fig. 4) to better understand the source of the transcription activity. Transcripts for endoglucanase genes (Fig. 4A), xylanase genes (Fig. 4B), and IL tolerance genes (Fig. 4F) appeared to come primarily from *Firmicutes*, particularly in the presence of IL. *Firmicutes* also played an important role in transcribing beta-glucosidase genes at 0.5% and 1% IL, while *Proteobacteria* were greatly involved at 0.5% IL (Fig. 4C). The transcription of cellulose 1,4-beta-cellobiosidase was very low compared to that of other enzymes and was seen primarily with *Actinobacteria* and *Firmicutes* (Fig. 4D). The elevated acetate kinase transcription observed in the 0.5% IL community was seen primarily with *Actinobacteria* (Fig. 4E).

**FIG 5:**
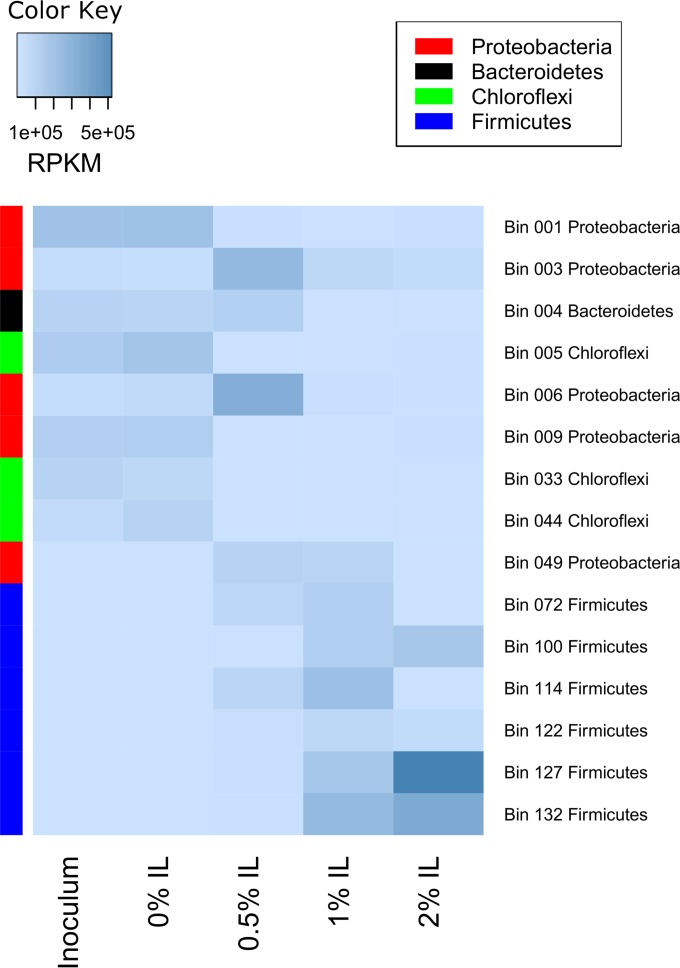
Highly transcribing microorganisms. The top 5 microorganisms producing high transcription levels at each IL concentration were pooled for further investigation, resulting in a list of 15 genomes. Darker colors indicate higher total transcription levels.

To understand the gene transcription profiles at the genome level, we selected the top five most highly transcribed genomes from each sample for further analysis. The top five genomes from each sample were merged into a single group of 15 genomes, and their transcription levels were compared across samples (Fig. 5). Although *Proteobacteria* exhibited robust transcription in both the 0% and 0.5% IL samples, the individual species that transcribed genes were not the same. For example, a *Proteobacteria* genome represented by bin 001 had the highest transcription levels in the inoculum and 0% IL samples; however, its gene expression levels decreased significantly in the sample with 0.5% IL. Another two proteobacterial genomes, represented by bins 003 and 006, did not display high transcription in the inoculum or 0% IL samples but showed increased transcription in the 0.5% IL sample, suggesting that these organisms persisted relative to other *Proteobacteria* in the 0.5% IL environment.

**FIG 6:**
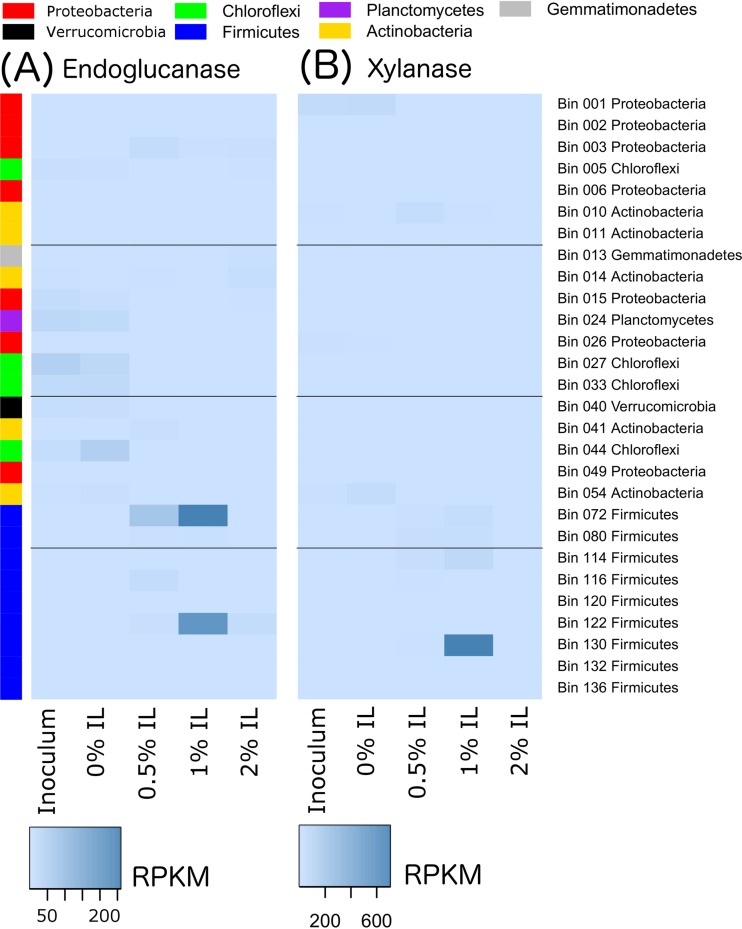
Transcription of cellulolytic genes disaggregated by genome. The top 3 genomes transcribing each gene class of interest at each IL concentration were pooled into a list of 28 genomes as shown on the right side of the heat maps. The phyla of the genomes are colored on the left side of the heatmaps for easier identification and are explained in the legend at the top of the figure. Transcription is shown for (A) endoglucanases and (B) xylanases.

**FIG 7:**
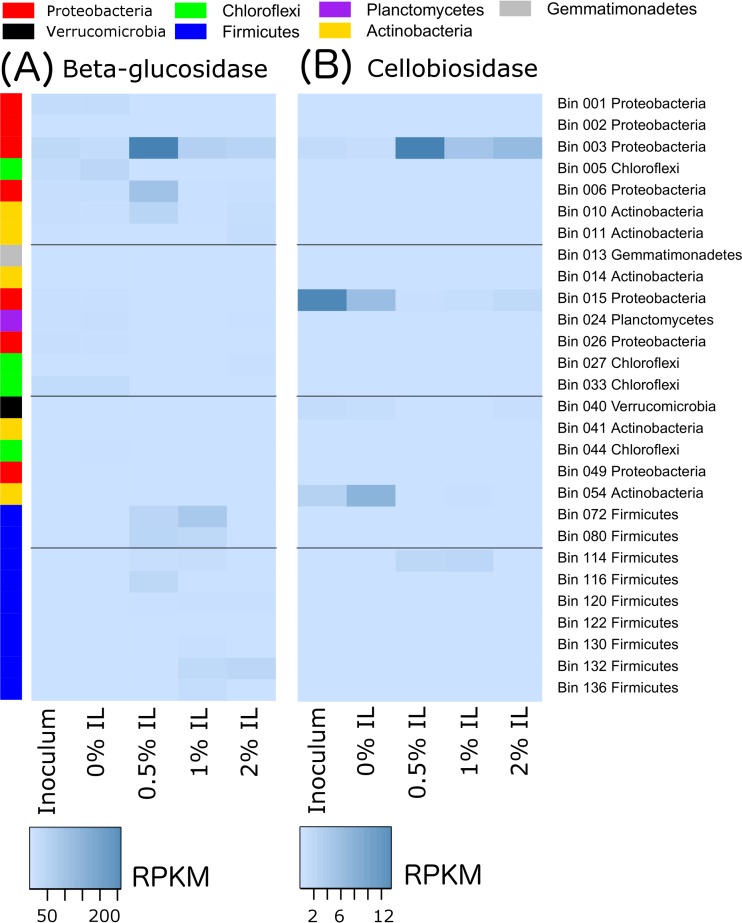
Transcription of cellulolytic genes disaggregated by genome. The top 3 genomes transcribing each gene class of interest at each IL concentration were pooled into a list of 28 genomes. The phyla of the genomes are colored on the left side of the heatmaps for easier identification and are explained in the legend at the top of the figure. Within this group, transcription is shown for (A) beta-glucosidases and (B) cellulose 1,4-beta-cellobiosidases.

**FIG 8:**
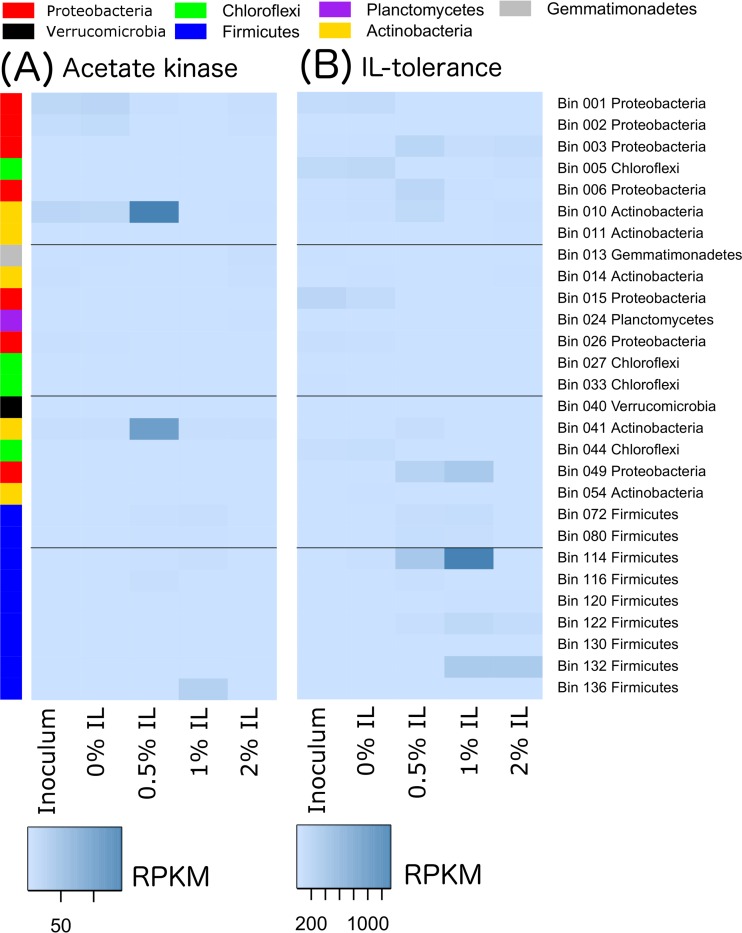
Transcription of acetate kinase and IL tolerance genes, disaggregated by genome, is shown for the same 28 genomes shown in Fig. 6. Data represent transcription of (A) acetate kinase genes and (B) IL tolerance genes.

We further collected the top three genomes that exhibited elevated transcription of six different gene classes of interest (endoglucanase, xylanase, cellulose 1,4-beta-cellobiosidase, beta-glucosidase, acetate kinase, and IL tolerance genes) for any sample, merged them into a 28-genome set, and plotted their transcription profiles (Fig. 6, 7, and 8). More-detailed organism identification information can be found in Table S2. As shown in Fig. 6A, endoglucanase transcripts were predominantly mapped to *Firmicutes* bins 72 (*Thermobacillus composti*, 85.5% identity) and 122 (*Brevibacillus thermoruber*, 93.1% identity) in the 1% IL sample. Xylanases were mostly transcribed by bin 130 at 1% IL (*Clostridium* sp., 66.3% identity), suggesting that they may act as a major hemicellulose degrader (Fig. 6B). Beta-glucosidases were most highly transcribed by bin 003 (*Pseudoxanthomonas suwonensis*, 82.4% identity) and bin 006 (*Luteimonas* sp. strain J29, 80.7% identity) at 0.5% IL as well as by bin 72 at 1% IL. It is worth noting that the organism represented by bin 003 also transcribed beta-glucosides at 1% and even 2% IL, although the amount was significantly lower than that seen at 0.5% IL. Cellulose 1,4-beta-cellobiosidase was the least transcribed enzyme among all cellulases, with the majority transcribed by *Proteobacteria* bin 015 (*Chelativorans* sp. strain J32, 81.7% identity) and *Actinobacteria* bin 054 (*Thermobifida fusca*, 83.9% identity) at 0% IL and bin 003 at 0.5% IL. Two *Actinobacteria* populations, as represented by bins 10 (*Streptosporangium roseum*, 74.4% identity) and 041 (*Mycobacterium hassiacum*, 99.7% identity), transcribed the highest level of acetate kinases in the 0.5% IL sample (Fig. 8A). Interestingly, these two species did not actively transcribe acetate kinases when the concentration of ionic liquid increased to 1% or 2%. Since the genome abundances of these two *Actinobacteria* significantly decreased with increasing IL concentration (those in bin 10 decreased from 168× to 29× and those in bin 41 decreased from 35× to 5× upon an increase of the IL concentration from 0.5% to 1%), they probably could not tolerate higher levels of ionic liquid.

Transcription of IL tolerance genes was also quantified for the recovered genomes, and the highest level of transcription was observed in the 1% IL sample from *Firmicutes* bin 114 (Fig. 8B). However, transcription by this organism decreased in the 2% IL sample, suggesting that it probably could not thrive at higher levels of ionic liquid as its genome coverage also decreased from 179× at 1% IL to 1× at 2% IL. *Firmicutes* bin 132 (*Bacillus thermoamylovorans*, 98.9%), on the other hand, also showed transcription of IL tolerance genes in the 1% IL sample but maintained transcription at 2% IL. Since its genome coverage increased from 144× at 1% IL to 328× at 2% IL, this species may be more tolerant of IL than the organism represented by bin 114.

### Analysis of *Firmicutes* Bin 127.

The population in bin 127, of the phylum *Firmicutes*, is distantly related to *Bacillus alveayuensis* (Table S2) and had the highest level of transcription in the 2% IL sample (Fig. 5). Interestingly, this population did not vigorously transcribe known biomass degradation genes or IL tolerance genes (Fig. 6, 7, and 8). Moreover, it did not highly transcribe acetate kinases, raising questions about its means of survival. By extracting the 5 most highly transcribed genes that mapped to this bin, we found that genes encoding one hypothetical protein, three putative transporters or permeases, and an isocitrate lyase were the genes with highest level of transcription at 2% IL (Table S7). Among the proportions of the total transcripts mapped to this bin, the isocitrate lyase gene alone accounted for 11.6%, 13.7%, and 7% of transcription in the 0.5%, 1%, and 2% IL samples, respectively (see Fig. S1 in the supplemental material). Upregulation of the genes encoding isocitrate lyase and malate synthase, two enzymes critical to the glyoxylate bypass, has been observed in *Escherichia coli* and *Corynebacterium glutamicum* during growth on acetate (16, 17). We did not find a highly expressed malate synthase gene in bin 127, probably because the genome is only ca. 60% complete. However, we hypothesize that the organism represented by bin 127 was able to utilize acetate obtained from the ionic liquid [C_2_mim][OAc] as its carbon source. The transcription of the three transporters may also be related to IL transport, although we did not identify the substrates that these transporters carry.

10.1128/mSystems.00120-16.8 Table S7  Top 5 most highly expressed genes in bin 127 for all five samples. Download Table S7, PDF file, 0.02 MB. Copyright © 2016 Wu et al. 2016 Wu et al. This content is distributed under the terms of the Creative Commons Attribution 4.0 International license.

10.1128/mSystems.00120-16.1 Figure S1  Gene transcription levels of the most highly transcribed genes for bin 127. The transcription-level percentages were calculated by dividing the transcription level of a single gene by the sum of the transcription levels for all genes. See Table S7 for the list of genes. Download Figure S1, TIF file, 0.5 MB. Copyright © 2016 Wu et al. 2016 Wu et al. This content is distributed under the terms of the Creative Commons Attribution 4.0 International license.

## DISCUSSION

Using metatranscriptomics with automated binning allowed us to track dynamic changes in a complex community at the transcriptome level and to do so with genome resolution. The presence of IL had a dramatic effect on the microbial community composition and transcription. The high-IL environment led to the dominance of members from a few phyla, primarily *Firmicutes* and, to a much lesser extent, *Proteobacteria*. Even within these phyla, fewer than a dozen genomes accounted for the majority of transcription activity, with members of the genera *Bacillus*, *Thermobacillus*, and *Brevibacillus* generating more than half of the transcription in the 1% and 2% IL communities. We acknowledge that there could have been RNA and DNA extraction biases that impacted these relative abundances due to differential extraction efficiency levels among different organisms (18). Nevertheless, the shift in relative abundance from *Proteobacteria* to *Firmicutes* under conditions of increasing IL was marked.

We hypothesize that organisms growing in the high-IL environment could survive in part due to their transcription of efflux pumps. Khudyakov et al. showed that osmoprotectant transporters and multidrug efflux pumps were upregulated in *Enterobacter lignolyticus* SCF1 in the presence of the ionic liquid, 1-ethyl-3-methylimidazolium chloride ([C_2_mim][Cl]) (9). Further study of this organism’s genes by Ruegg et al. revealed that a single multidrug efflux pump (*eilA*) was entirely responsible for the IL tolerance of SCF1 (13). When its gene was cloned into *E. coli*, this pump was shown to primarily export quaternary ammonium cations, including 1-ethyl-3-methylimidazolium. Homologous pumps have been observed in other enterobacteria and have likewise been shown to confer resistance to quaternary ammonium toxins such as methyl viologen (19, 20). In *Bacilli*, the small multidrug resistance proteins (SMR) encoded by the genes *ykkC* and *ykkD* have been shown to confer resistance to hydrophobic quaternary amines, including ethidium bromide, methyl viologen, crystal violet, pyronin Y, and cetylpyridinium chloride (21). We observed homologues to these genes in our metatranscriptomic analysis and hypothesize that they play a role in resistance to [C_2_mim][OAc]. Our findings of strong [C_2_mim][OAc] resistance in *Bacilli* are consistent with findings by Simmons et al. in which *Bacillus coagulans* was found to exhibit tolerance of 4% [C_2_mim][Cl] and [C_2_mim][OAc] (22).

Interestingly, we observed higher rates of cellulolytic enzyme transcription under conditions in which the IL concentration was increased to 1%. This was likely due to the selection of *Bacilli* that not only tolerate IL but also highly express cellulolytic enzymes. Indeed, xylanase enzyme activity was highest in 1% IL communities, validating the transcriptomic results. Endoglucanase enzyme activity resulted in a less clear trend. Nevertheless, a high level of endoglucanase activity could be achieved in up to 1% IL, suggesting that the moderately IL-tolerant organisms can also be effective cellulose degraders.

Given the very low level of cellulolytic enzyme transcription in the 2% IL community, we were surprised by the relatively high respiration level of these cultures. Moreover, this finding was corroborated by high total transcription levels in this community. These results led us to consider whether organisms were consuming the acetate anion from the ionic liquid. We investigated acetate kinase activity in the 2% community, but found that it was almost nonexistent. Moreover, the organisms expressing acetate kinase (primarily *Actinobacteria*) were among the least active transcribers in the community. Further investigation of upregulated genes within the most dominant *Firmicutes* bin (bin 127) revealed dramatic upregulation of isocitrate lyase, a gene critical to the glyoxylate bypass. In other organisms, upregulation of this gene has been observed in the presence of acetate (16, 17) and is likely involved in acetate metabolism. Three putative transporters in this bin also had very high transcription levels, and we hypothesize that they are responsible for ionic liquid transport, but further characterization is required to provide validation. While we hypothesize that this organism was utilizing the abundant acetate from the ionic liquid, the exact mechanisms of acetate metabolism and strong IL tolerance merit further study.

In addition to showing that communities of organisms can be simultaneously IL tolerant and effective (hemi)cellulose degraders, we were able to identify specific organisms that show promise for industrial applications. Specifically, the *Firmicutes* corresponding to bins 72, 122, and 130 were all active at 1% IL and were among the most active transcribers of cellulases and xylanases. The enzymes transcribed by these organisms merit more-detailed study to better understand their tolerance of IL and its effects on their binding and catalytic activities. Conversely, the IL tolerance genes that were upregulated in the 1% IL community did not consistently appear in the 2% community. This suggests that either the tolerance of these organisms was limited in spite of efflux pump gene transcription or some of these genes were not in fact involved in IL tolerance. Here too, follow-up cloning studies should reveal the specific function and effectiveness of these genes in conferring IL tolerance. This knowledge could provide new tools for engineering organisms to function on IL-treated biomass.

## MATERIALS AND METHODS

### Incubations with high levels of solids.

Switchgrass (*Panicum virgatum L.*) was obtained from the Joint Bioenergy Institute (Emeryville, CA). Biomass was size reduced using a leaf shredder and air-dried until the moisture level was <10%. It was further size reduced using a Wiley mill with a 10-mm-pore-size screen. Switchgrass was stored in airtight containers at 4°C until needed. The inoculum was obtained from a community enriched with switchgrass under thermophilic conditions with high solid concentrations (23).

High-solid incubations were completed with various concentrations of 1-ethyl-3-methylimidazolium acetate ([C_2_mim][OAc]) amended to switchgrass on a percent dry weight basis (0%, 0.5%, 1%, and 2% IL). Incubations were conducted as described previously (14, 24). In summary, switchgrass was wetted with minimal media (25) to a moisture content of 400% (wt) dry basis (g water [g dry solid]^−1^), inoculated with 10% (wt) inoculum (g dry inoculum [g dry solid]^−1^), and then amended with IL. Bioreactors with a working volume of 0.2 liters were loaded with the switchgrass mixture. Samples were aerated and incubated for 1 week at 55°C. Effluent from each reactor was monitored using an infrared carbon dioxide (CO_2_) sensor (Vaisala, Woburn, MA), and flow was measured with a thermal mass flowmeter (Aalborg, Orangeburg, NY). Carbon dioxide and flow data were recorded by LabVIEW. The experiment was conducted with three replicates.

### Enzyme extraction from solid samples.

Enzymes were extracted from final feedstocks with a buffer containing 1% (wt) sodium chloride, 0.1% (wt) Tween 80, and 50% (wt) ethylene glycol (24). Freshly collected final feedstock (3 g wet weight) was shaken with 27 g of buffer for 1 h at 150 rpm and ambient temperature. Samples were centrifuged at 4°C and 10,000 × *g* for 20 min and then vacuum filtered using 0.2 µm-pore-size membranes. The extraction buffer was exchanged with sodium acetate buffer (50 mM, pH 5.0) using Vivaspin columns (VWR, West Chester, PA) with a polyethersulfone (PES) membrane and a molecular weight cutoff value of 5. Endoglucanase and xylanase activities in dialyzed extracts were measured as described previously (24). All enzyme extractions and assays were completed in triplicate. Activities were reported as international units (IU)·per gram of dry matter where 1 IU = 1 μmol product min^−1^.

### DNA and total RNA extraction from solid samples.

Following incubation with high solid concentrations, 2-g samples of biomass were frozen in liquid nitrogen and homogenized with a Retsch MM400 oscillating ball mill (Verder Scientific, Inc., Newtown, PA). Microbial RNA was stabilized with LifeGuard Soil Preservation solution (Mo Bio Laboratories, Inc., Carlsbad, CA) at a ratio of 1:2.5 (sample/LifeGuard solution), and samples were stored at −80°C. Samples were subsequently thawed on ice, the LifeGuard solution was removed by centrifugation, and solids were processed with a PowerSoil total RNA isolation kit (Mo Bio, Inc.). Samples were treated with 25 µl 2-mercaptoethanol to denature RNases, and then the Mo Bio, Inc., kit protocol was used to isolate nucleic acids. After eluting RNA from the anion exchange column, DNA was recovered using a Mo Bio, Inc., DNA elution accessory kit. DNA digestion and cleanup of the RNA samples were achieved using an RNase-free DNase set and RNeasy minikit (Qiagen, Valencia, CA), respectively. A Joint Genome Institute (JGI) Sequencing Technology protocol was used for DNase treatment (26), followed by the use of the Qiagen kit protocol for cleanup (Qiagen, Valencia, CA). RNA samples of each type were pooled, concentrated, and further purified using an RNeasy minikit. DNA samples of each type were pooled and diluted with elution buffer. RNA quality was assessed with a model 2100 Bioanalyzer (Agilent Technologies, Santa Clara, CA), a Qubit fluorometer (Life Technologies, Inc., Grand Island, NY), and a NanoDrop 2000 spectrophotometer (Thermo Scientific, Wilmington, DE). DNA and total RNA samples were submitted to the Joint Genome Institute for cDNA synthesis and high-throughput sequencing using an Illumina HiSeq 2000 system. For RNA sequencing, 1 μg total RNA sample was treated with Ribo-Zero rRNA removal kits (Epicentre, Madison, WI). The treated RNA sample was reacted with Ribo-Zero magnetic beads, and the rRNA-depleted RNA was collected. Purification was done by mixing and incubating rRNA-depleted RNA samples with 160 μl AMPure XP beads (Agencourt Bioscience, Beverly, MA) at room temperature for 5 min, washing with 75% ethanol, drying the beads, and then mixing with 11 μl elution buffer. Prior to fragmentation, purified sample was checked with an mRNA Pico Chip to ensure that the mRNA sample had less than 5% rRNA.

cDNA was synthesized from fragmented mRNA using SuperScript II reverse transcriptase (Invitrogen, Carlsbad, CA) according to the manufacturer’s guidelines. In brief, SuperScript II (Invitrogen, Carlsbad, CA) was used as a primer during first-strand synthesis. The resultant double-stranded cDNA was mixed with resuspension buffer and a-tailing mix and processed to ligate adapters. Once the ligation reaction and cleaning were completed, cDNA was enriched via 10 cycles of PCR with Illumina TruSeq primers (Illumina, San Diego, CA).

### Data analysis.

Respiration data from incubations with high solid concentrations were used to calculate CO_2_ evolution rates (CER) and cumulative CO_2_ respiration rates (cCER) from CO_2_ concentrations and volumetric flow rate measurements of reactor effluents, as described previously (27). CER values were normalized according to the dry weight of material in the reactor. cCER values were obtained by integrating CER over time.

The R statistical package was used to perform analysis of variance (ANOVA) and Tukey’s honestly significant difference (HSD) test on data obtained from respiration studies and enzyme assays. Specifically, the “car” and “agricolae” packages were run within R to carry out these analyses.

Sequences obtained through high-throughput sequencing of isolated DNA were quality trimmed, filtered, assembled, and assigned to operational taxonomic units (OTUs) using methods described previously (28). 16S rRNA gene read counts were used to conduct ecological and ordination analyses. Singletons were removed to reduce variability. Operational taxonomic unit (OTU) richness and evenness values and Shannon diversity values were computed in R (version 3.0.2; R Foundation for Statistical Computing, Vienna, Austria; https://www.r-project.org/) using the Vegan package, which was downloaded within R using a mirror site. Significant differences between treatments were identified using ANOVA and least significant difference with a significance level α = 0.05. Data were analyzed using SAS software (Version 9.4; SAS Institute, Cary, NC).

### Metagenomics assembly and individual genome recovery (binning).

To coassemble the five individual metagenomes, sequencing reads of the enriched switchgrass microbial community and of the four communities amended with different ionic liquid concentrations were trimmed using Trimmomatics (ILLUMINACLIP:TruSeq3-PE.fa:2:30:10 LEADING:3 TRAILING:3 SLIDINGWINDOW:4:15 MINLEN:36) and coassembled using IDBA-UD (29) with the “–pre_correction” option. The assembled scaffolds were then binned using MaxBin 2.0 (15, 30) with default settings (minimum length cutoff, 1,000 bp; minimum probability threshold, 0.9) to recover individual genomes. Genes were predicted using Prodigal (31) with the “-p meta” option for metagenomic sequence annotation and the “-f gff -o output.gff -a output.faa” option to output the GFF file along with the predicted amino acid sequences. The species with genomes closest to the recovered genomes were determined by (i) searching the predicted genes against the nonredundant (NR) protein database using RAPSearch2 (32); (ii) checking the taxonomy of the closest hits for all genes; and (iii) assigning the taxonomy of the most frequently appearing taxonomy to each genome. Amino acid identity was also determined by averaging the amino acid identities of the most frequently appearing of the closest hits from the RAPSearch2 results for each genome. Bins assigned to any taxonomy that belonged to eukaryotes were noted in the phylum assignment only as “(Eukaryotes)” but were not involved in downstream metatranscriptomics analysis due to the differences between prokaryotes and eukaryotes with respect to gene structure. Scaffolds not assigned to any bin—due either to the minimum length limit or to the probability cutoff of MaxBin 2.0—were searched against the NCBI nonredundant (NR) database using DIAMOND (33) with the BLASTX option. The resulting data file was then converted to tab format using the VIEW option of DIAMOND and then input into MEGAN (34) to get the phylum-level assignment of the scaffolds. Due to its lowest-common-ancestor (LCA) algorithm, MEGAN assigns the scaffolds to the highest common taxonomic levels based on the taxonomic distribution of the BLASTP hits of each scaffold. Scaffolds assigned to “eukaryotes,” “cellular organisms,” or “unassigned sequences” were not pursued in downstream metatranscriptomic analysis; those assigned to the “*Bacteria*” kingdom were annotated as “unclassified bacteria.”

### Annotation of cellulose degradation genes.

The KEGG (Kyoto Encyclopedia of Genes and Genomes) orthologs (KO) of the genes were identified using HMMER3 (35) by comparing the predicted genes to those in the file FOAM-hmm_rel1.hmm that came with FOAM (36). The hmm files that were produced were parsed using the scripts and instructions provided with FOAM {sort output.hmm > output.sort; python bmn-HMMerBestHit.py output.sort > output.BH; awk --F “ “ ‘(print $1,”\t”,$4)’ output.BH | sed s/[\ ]*KO:// > output.KO}.

The EC annotations were also extracted by searching the genes in the coassembled scaffolds using BLASTP against the five individual metagenomes assembled and annotated by the Joint Genome Institute, which designated the EC numbers by searching genes in an isolated genome database using USEARCH (37). Only genes that can be mapped to the JGI-annotated genes with at least 95% identity and 40% coverage were assigned the corresponding EC number.

Both the KEGG annotations and EC numbers were used to identify genes that can be classified into categories of genes encoding five different enzyme types, which were endoglucanases (K01179, K19356, K19357, and K20542; EC 3.2.1.4), cellulose 1,4-beta-cellobiosidases (K01225 and K19668; EC 3.2.1.91), beta-glucosidases (K01188, K05349, and K05350; EC 3.2.1.21), xylanases (K01181 and K13465; EC 3.2.1.8), and acetate kinases (K00925; EC 2.7.2.1). A cross comparison between the KO and EC annotations for the five classes of enzymes showed that the five FOAM-annotated enzyme classes were generally in agreement with the EC annotations, except for cellulose 1,4-beta-cellobiosidases, for which only 14% of the FOAM annotations matched the EC number (Table S8). As a result, both the KEGG and EC annotations for any enzyme type need to be matched in order to classify genes into specific enzymes, except for cellulose 1,4-beta-cellobiosidase, for which only the EC numbers need to be used to identify members of this class of enzyme due to its low KEGG/EC-matching ratio. In other words, genes with inconsistent KEGG and EC annotations (for example, a K01179 gene with EC no. 3.2.1.91) were not considered.

10.1128/mSystems.00120-16.9 Table S8  Percentages of KO annotations matched to EC numbers. Download Table S8, PDF file, 0.02 MB. Copyright © 2016 Wu et al. 2016 Wu et al. This content is distributed under the terms of the Creative Commons Attribution 4.0 International license.

### Annotation of ionic liquid tolerance genes.

Genes demonstrated to confer ionic liquid (IL) tolerance, including *Enterobacter lignolyticus eilA* (locus tag Entcl_2352) (13), *E. coli* K-12 *sugE* (locus tag b4148) and *emrE* (locus tag b0543) (38, 39), *Salmonella enterica smvA* (locus tag STM1574) (40, 41), and *Bacillus cereus ykkC* and *ykkD* (21), were downloaded, from the NCBI website (http://www.ncbi.nlm.nih.gov/). The predicted genes were searched against the downloaded IL tolerance genes using BLASTP with the *E* value cutoff set to 1e-5.

### Metatranscriptome analysis.

Metatranscriptomic reads were trimmed using Trimmomatic (ILLUMINACLIP:TruSeq3-PE.fa:2:30:10 LEADING:3 TRAILING:3 SLIDINGWINDOW:4:15 MINLEN:36) (42). The trimmed paired-end reads were then aligned with the coassembled metagenomic scaffolds using Bowtie2 (43) with default options using 8 threads. HTSeq (44) and the GFF file produced by Prodigal were used to parse the produced SAM files to get the read count for the predicted genes, and the rpkm() function of edgeR (45) was employed (with gene lengths calculated by a customized Perl script) to obtain the counts of normalized reads per kilobase per million reads (RPKM) for the genes. Binned eukaryotic genomes and sequences classified as “eukaryotes,” “cellular organisms,” or “unassigned” by MEGAN were excluded from the metatranscriptomic analysis since eukaryotic genes are structurally different from prokaryotic genes and since improperly predicted genes greatly alter the quantification of gene expression levels in terms of RPKM, as the RPKM metric normalized against both to the data set sizes and the gene lengths.

### Data archiving.

The metagenome and metatranscriptome raw reads, assembled scaffolds, and gene annotations can be accessed through IMG (http://img.jgi.doe.gov/). The metagenomes are listed as Taxon Object identifiers (IDs) 3300002523 (inoculum), 3300002531 (0% IL), 3300002542 (0.5% IL), 3300002541 (1% IL), and 3300002559 (2% IL) and metatranscriptomes as Taxon Object IDs 3300003330 (inoculum), 3300003331 (0% IL), 3300003329 (0.5% IL), 3300003327 (1% IL), and 3300003326 (2% IL).
