# Supplementary material for: Ionic Liquids Impact the Bioenergy Feedstock-Degrading Microbiome and Transcription of Enzymes Relevant to Polysaccharide Hydrolysis
Source: mSystems. 2016 Dec 13;1(6):e00120-16. doi: 10.1128/mSystems.00120-16 (PMC5155067; doi:10.1128/mSystems.00120-16)
Supplement: Table S1 [file sys006162071st1.pdf]

Table S1. Data set sizes of the 16S amplicons, metagenomes, and metatranscriptomes

Samples	16S amplicon	Metagenome	Metatranscriptome
Inoculum		17G bps	29G bps
0% IL		25G bps	15G bps
0.5% IL		19G bps	26G bps
1% IL		25G bps	12G bps
2% IL		28G bps	24G bps
