# Supplementary material for: Ionic Liquids Impact the Bioenergy Feedstock-Degrading Microbiome and Transcription of Enzymes Relevant to Polysaccharide Hydrolysis
Source: mSystems. 2016 Dec 13;1(6):e00120-16. doi: 10.1128/mSystems.00120-16 (PMC5155067; doi:10.1128/mSystems.00120-16)
Supplement: Table S2 [file sys006162071st2.pdf]

Table S2. Taxonomic and genome information of the recovered genome bins.

Bin	Phylum	Closest species (amino acid identity)	Completeness[a]	Genome size	GC %
001	Proteobacteria	*Thioalkalivibrio sulfidophilus* (64.06%)	94.40%	3475536	64.1
002	Proteobacteria	*Sphingobium* sp. SYK-6 (64.93%)	95.30%	3665541	67.7
003	Proteobacteria	*Pseudoxanthomonas suwonensis* (82.38%)	83.20%	2502625	72.5
004	Bacteroidetes	*Niabella aurantiaca* (72.92%)	84.10%	2984673	40.7
005	Chloroflexi	*Anaerolinea thermophila* (58.78%)	90.70%	4812602	60.8
006	Proteobacteria	*Luteimonas* sp. J29 (80.66%)	95.30%	2820705	69.8
007	Planctomycetes	*Phycisphaera mikurensis* (60.68%)	90.70%	3989218	70.6
008	Proteobacteria	*Chelatococcus* sp. GW1 (90.67%)	91.60%	3208607	68.1
009	Proteobacteria	*Caldimonas manganoxidans* (79.51%)	99.10%	3870094	70.3
010	Actinobacteria	*Streptosporangium roseum* (74.40%)	83.20%	4884654	72.6
011	Actinobacteria	*Candidatus* Actinomarina minuta (60.11%)	88.80%	2562596	69.3
012	(unclassified) [b]	*Candidatus* Saccharibacteria bacterium GW2011_GWA2_46_10 (65.16%)	56.10%	1081956	53.5
013	Gemmatimonadetes	Gemmatimonadetes bacterium KBS708 (60.34%)	93.50%	4038678	70.9
014	Actinobacteria	*Mycobacterium thermoresistibile* (80.84%)	90.70%	4017704	72
015	Proteobacteria	*Chelativorans* sp. J32 (81.72%)	87.90%	4187552	63.1
016	Actinobacteria	*Thermomonospora curvata* (99.61%)	97.20%	5373478	71.8
017	Actinobacteria	*Solirubrobacterales* bacterium URHD0059 (73.87%)	89.70%	2464499	73.1
018	Acidobacteria	*Candidatus* Solibacter usitatus (61.23%)	93.50%	3266445	69.4
019	Actinobacteria	*Chelativorans* sp. J32 (77.62%)	96.30%	4498608	62.6
020	(Eukaryote) [c]			531264	32.8
021	Proteobacteria	*Filomicrobium* sp. W (69.74%)	83.20%	3630912	60.5
022	Actinobacteria	*Conexibacter woesei* (65.02%)	87.90%	2682778	73
023	Proteobacteria	*Alkalilimnicola ehrlichii* (68.99%)	99.10%	4011724	68.6
024	Planctomycetes	*Pirellula staleyi* (62.55%)	92.50%	6004265	64

025	Proteobacteria	*Nitrosococcus halophilus* (60.31%)	92.50%	4628292	61.8
026	Proteobacteria	*Nitrosococcus halophilus* (60.28%)	98.10%	3400277	62.1
027	Chloroflexi	*Roseiflexus* sp. RS-1 (72.01%)	75.70%	6073077	71.2
028	Proteobacteria	*Sorangium cellulosum* (61.99%)	95.30%	8172849	69.9
029	Actinobacteria	*Candidatus* Microthrix parvicella (63.27%)	85.00%	3310956	75.6
030	Actinobacteria	*Ilumatobacter coccineus* (68.40%)	93.50%	3893026	72.7
031	Chloroflexi	*Sphaerobacter thermophilus* (64.36%)	93.50%	4746850	65.8
032	Deinococcus-Thermus	*Truepera radiovictrix* (66.43%)	68.20%	2880669	73.6
033	Chloroflexi	*Roseiflexus* sp. RS-1 (68.69%)	98.10%	6996601	58.8
034	Planctomycetes	*Phycisphaera mikurensis* (59.06%)	93.50%	4093275	64.1
035	Acidobacteria	*Candidatus* Solibacter usitatus (65.07%)	81.30%	5502542	58.9
036	Actinobacteria	*Conexibacter woesei* (77.68%)	84.10%	2800399	76.2
037	Proteobacteria	*Rhodospirillales* bacterium URHD0088 (68.10%)	98.10%	4336571	65.9
038	Actinobacteria	*Patulibacter sp*. (53.10%)	5.60%	414106	66.1
039	Actinobacteria	*Micromonospora carbonacea* (76.79%)	99.10%	5394202	72.7
040	Verrucomicrobia	*Chthoniobacter flavus* (63.07%)	90.70%	3571856	62.5
041	Actinobacteria	*Mycobacterium hassiacum* (99.73%)	94.40%	5323450	69.4
042	Actinobacteria	*Aeromicrobium marinum* (64.20%)	2.80%	426906	63.4
043	Actinobacteria	*Thermobifida fusca* (89.70%)	4.70%	683055	64.6
044	Chloroflexi	*Oscillochloris trichoides* (64.28%)	94.40%	7806912	54.3
045	Firmicutes	*Symbiobacterium thermophilum* (61.91%)	98.10%	2949845	67.4
046	Proteobacteria	*Belnapia moabensis* (75.12%)	70.10%	3974916	73
047	Firmicutes	*Paenibacillus ginsengihumi* (71.04%)	77.60%	4061821	57.5
048	Actinobacteria	*Conexibacter woesei* (65.63%)	90.70%	3431598	71
049	Proteobacteria	*Bordetella petrii* (79.71%)	100.00%	3555599	66.9
050	Actinobacteria	*Dactylosporangium aurantiacum* (69.62%)	67.30%	4697130	71.9
051	Proteobacteria	*Bordetella petrii* (70.47%)	97.20%	2897843	63
052	Proteobacteria	*Inquilinus limosus* (68.38%)	89.70%	4573808	70.9

053	Proteobacteria	*Azospira oryzae* (69.66%)	98.10%	2754323	66.6
054	Actinobacteria	*Thermobifida fusca* (83.85%)	72.90%	4366661	72.3
055	Gemmatimonadetes	Gemmatimonadetes bacterium KBS708 (62.89%)	13.10%	348348	69.9
056	Proteobacteria	*Filomicrobium* sp. W (69.45%)	92.50%	6047331	61.4
057	Gemmatimonadetes	Gemmatimonadetes bacterium KBS708 (63.29%)	0.00%	550887	72.9
058	Proteobacteria	*Nitrosococcus halophilus* (58.61%)	96.30%	4705754	61.8
059	Proteobacteria	*Bdellovibrio bacteriovorus* (59.65%)	98.10%	3221967	54
060	Actinobacteria	*Jiangella alkaliphila* (67.19%)	87.90%	3885222	74.6
061	Proteobacteria	*Bordetella petrii* (73.79%)	86.90%	4177445	65.4
062	Proteobacteria	*Bordetella hinzii* (88.78%)	10.30%	2327317	58.6
063	Actinobacteria	*Conexibacter woesei* (66.28%)	52.30%	2633280	74.5
064	Planctomycetes	*Phycisphaera mikurensis* (62.04%)	95.30%	3503091	62.2
065	Proteobacteria	*Methylocaldum szegediense* (64.91%)	81.30%	4784654	67.4
066	Proteobacteria	*Kordiimonas gwangyangensis* (66.24%)	96.30%	3271114	66.3
067	Actinobacteria	*Conexibacter woesei* (65.56%)	95.30%	2054099	73.6
068	Actinobacteria	*Conexibacter woesei* (68.38%)	62.60%	3463371	75.4
069	Actinobacteria	*Thermomonospora curvata* (100.00%)	0.00%	307003	68.8
070	Gemmatimonadetes	Gemmatimonadetes bacterium KBS708 (60.99%)	62.60%	3768004	70.9
071	Gemmatimonadetes	Gemmatimonadetes bacterium KBS708 (60.81%)	95.30%	3775846	70.5
072	Firmicutes	*Thermobacillus composti* (85.45%)	90.70%	4670286	52.5
073	Proteobacteria	Alpha proteobacterium JLT2015 (63.50%)	96.30%	3163780	67.4
074	Actinobacteria	*Thermobispora bispora* (98.23%)	56.10%	4278955	71.5
075	Planctomycetes	*Phycisphaera mikurensis* (56.83%)	90.70%	5808029	65.6
076	Proteobacteria	*Dongia* sp. URHE0060 (67.84%)	86.00%	4029430	67
077	Actinobacteria	*Dactylosporangium aurantiacum* (71.26%)	56.10%	4497961	71.9
078	Firmicutes	Clostridiales bacterium PH28_bin88 (59.81%)	96.30%	5949782	66.5
079	Actinobacteria	*Solirubrobacter soli* (79.83%)	56.10%	3100520	71.4
080	Firmicutes	*Thermobacillus composti* (97.90%)	85.00%	3685388	62

081	Chloroflexi	*Sphaerobacter thermophilus* (98.63%)	70.10%	6194966	68
082	Verrucomicrobia	*Chthoniobacter flavus* (65.84%)	84.10%	3508425	59
083	Gemmatimonadetes	Gemmatimonadetes bacterium KBS708 (60.07%)	86.90%	6889575	66.4
084	Actinobacteria	*Pseudonocardia dioxanivorans* (75.84%)	96.30%	6800216	73
085	Actinobacteria	*Solirubrobacter soli* (74.63%)	17.80%	3171396	74.8
086	Proteobacteria	*Chelativorans* sp. J32 (79.09%)	15.90%	5891029	56.3
087	(Eukaryote)			9375488	47.2
088	Actinobacteria	*Streptosporangium roseum* (72.39%)	15.90%	4466683	75.2
089	Firmicutes	*Geobacillus thermodenitrificans* (99.57%)	97.20%	5252116	47.5
090	Actinobacteria	*Conexibacter woesei* (64.10%)	60.70%	2924126	71.8
091	Firmicutes	*Thermobacillus composti* (77.65%)	27.10%	1454691	63
092	Firmicutes	*Thermobacillus composti* (81.95%)	63.60%	2691226	59.1
093	Firmicutes	*Cohnella laeviribosi* (72.51%)	25.20%	690129	64.7
094	Proteobacteria	*Chelativorans* sp. J32 (78.42%)	39.30%	4926609	62.1
095	Proteobacteria	*Sorangium cellulosum* (64.14%)	80.40%	6077248	67.3
096	Actinobacteria	Marine actinobacterium MedAcidi-G2B (64.65%)	92.50%	4855510	70.9
097	Proteobacteria	*Bdellovibrio bacteriovorus* (60.36%)	83.20%	3053639	51.9
098	Firmicutes	*Thermobacillus composti* (77.91%)	5.60%	956669	67.3
099	Firmicutes	*Thermobacillus composti* (75.41%)	42.10%	2418936	60.5
100	Firmicutes	*Bacillus smithii* (98.41%)	72.90%	2463437	40.1
101	Firmicutes	*Bacillus thermoamylovorans* (73.66%)	98.10%	8100266	41.2
102	(Eukaryote)			19527087	53.5
103	Chloroflexi	*Oscillochloris trichoides* (70.71%)	68.20%	5738301	72.8
104	Firmicutes	*Thermobacillus composti* (79.28%)	25.20%	2731397	53.1
105	Proteobacteria	*Oceanibaculum indicum* (65.75%)	75.70%	4034044	63
106	Verrucomicrobia	*Pedosphaera parvula* (60.73%)	69.20%	3894320	62.8
107	(Eukaryote)			23851678	35.7
108	(Eukaryote)			735159	60.6

109	(Eukaryote)			8110372	48
110	(Eukaryote)			1329684	63.9
111	Chloroflexi	*Sphaerobacter thermophilus* (93.70%)	73.80%	4433798	68.4
112	(Eukaryote)			812611	56.8
113	(Eukaryote)			859691	56.5
114	Firmicutes	*Thermobacillus composti* (77.10%)	97.20%	3421115	53.2
115	Planctomycetes	*Chthonomonas calidirosea* (61.68%)	51.40%	3608883	66.8
116	Firmicutes	*Thermobacillus composti* (87.58%)	68.20%	3497721	64.6
117	Firmicutes	*Bacillus thermoamylovorans* (92.80%)	12.10%	5610467	32
118	Proteobacteria	*Inquilinus limosus* (71.99%)	96.30%	4532508	67.2
119	Firmicutes	*Thermobacillus composti* (77.77%)	97.20%	4323597	58
120	Firmicutes	*Cohnella laeviribosi* (99.07%)	96.30%	4308453	57.7
121	Firmicutes	*Thermobacillus composti* (87.11%)	66.40%	3755183	63.5
122	Firmicutes	*Brevibacillus thermoruber* (93.14%)	94.40%	3863006	56.4
123	Actinobacteria	*Thermobifida fusca* (99.85%)	88.80%	3713674	67.5
124	Actinobacteria	*Dactylosporangium aurantiacum* (73.64%)	66.40%	1804394	71.7
125	Bacteroidetes	*Niastella koreensis* (73.53%)	69.20%	6057648	47.7
126	Firmicutes	*Brevibacillus panacihumi* (78.49%)	97.20%	3731259	54.9
127	Firmicutes	*Bacillus alveayuensis* (78.86%)	59.80%	1589753	39.2
128	Firmicutes	*Tuberibacillus calidus* (98.83%)	89.70%	3411171	43.7
129	Firmicutes	*Thermobacillus composti* (79.48%)	97.20%	3930229	58.3
130	Firmicutes	Clostridium sp. KNHs205 (66.32%)	95.30%	4937026	36.3
131	Firmicutes	*Paenibacillus* sp. UNC451MF (61.65%)	93.50%	5378712	53
132	Firmicutes	*Bacillus thermoamylovorans* (98.85%)	95.30%	4581459	37.4
133	Firmicutes	*Ureibacillus thermosphaericus* (87.05%)	99.10%	4466128	39.7
134	Firmicutes	*Paenibacillus* sp. UNC451MF (68.48%)	98.10%	4167101	50.2
135	Firmicutes	*Paenibacillus ginsengihumi* (99.22%)	99.10%	5772385	57.5
136	Firmicutes	*Caldibacillus debilis* (98.62%)	97.20%	3387941	51.1

137	Firmicutes	*Tuberibacillus calidus* (73.41%)	96.30%	3447197	51.4
138	Firmicutes	*Bacillus coagulans* (99.53%)	80.40%	2803041	46.7

[a] Completeness was estimated by calculating the proportion of 107 single copy marker genes, which can be found in 95% of bacteria, that uniquely appear in each recovered genome.

[b] Here the phylum of this recovered genome is undetermined since its closest species, *Candidatus* Saccharibacteria bacterium, still do not have a phylum level taxonomic annotation.

[c] We did not attempt to resolve the recovered eukaryotic genomes since MaxBin was not designed to recover eukaryotic species, and the genomes of the eukaryotes may be highly fragmented. Completeness is also not measured for all recovered eukaryotic genomes. These eukaryotic genomes are also not involved in any downstream metatranscriptomics analysis.
