# Supplementary material for: Ionic Liquids Impact the Bioenergy Feedstock-Degrading Microbiome and Transcription of Enzymes Relevant to Polysaccharide Hydrolysis
Source: mSystems. 2016 Dec 13;1(6):e00120-16. doi: 10.1128/mSystems.00120-16 (PMC5155067; doi:10.1128/mSystems.00120-16)
Supplement: Table S3 [file sys006162071st3.pdf]

Table S3. The coverages and relative abundance ratios estimated for the genomes recovered from the metagenomes

		Genome coverages					Relative abundance ratio (%)				
Bin	Phylum	Inoculum	0% IL	0.5% IL	1% IL	2% IL	Inoculum	0% IL	0.5% IL	1% IL	2% IL
001	Proteobacteria	852.37	875.62	47.89	43.48	35.27	21.5%	21.0%	0.7%	1.4%	1.4%
002	Proteobacteria	338.81	282.21	13.8	15.5	39.47	8.5%	6.8%	0.2%	0.5%	1.6%
003	Proteobacteria	330.7	244.62	2469.76	657.27	147.74	8.3%	5.9%	38.1%	21.1%	5.9%
004	Bacteroidetes	187.66	272.17	121.87	8.6	8.52	4.7%	6.5%	1.9%	0.3%	0.3%
005	Chloroflexi	176.39	288.52	10.94	10.96	20.65	4.4%	6.9%	0.2%	0.4%	0.8%
006	Proteobacteria	130.7	187.93	1057.51	75.75	15.18	3.3%	4.5%	16.3%	2.4%	0.6%
007	Planctomycetes	119.4	216.43	5.31	6.21	14.84	3.0%	5.2%	0.1%	0.2%	0.6%
008	Proteobacteria	112.79	90.84	193.05	37.25	17.2	2.8%	2.2%	3.0%	1.2%	0.7%
009	Proteobacteria	102.23	148.69	25.47	19.35	18.11	2.6%	3.6%	0.4%	0.6%	0.7%
010	Actinobacteria	95.26	94.13	168.34	29.41	9.39	2.4%	2.3%	2.6%	0.9%	0.4%
011	Actinobacteria	94.6	46.84	6.54	6.61	19.43	2.4%	1.1%	0.1%	0.2%	0.8%
012	(unclassified)	83.3	9.81	4.09	3.95	10.45	2.1%	0.2%	0.1%	0.1%	0.4%
013	Gemmatimonadetes	82.13	79.31	1.19	1.29	4.31	2.1%	1.9%	0.0%	0.0%	0.2%
014	Actinobacteria	78.01	64.68	27.31	13.14	17.1	2.0%	1.5%	0.4%	0.4%	0.7%
015	Proteobacteria	72.05	71.28	5.01	4.17	9.52	1.8%	1.7%	0.1%	0.1%	0.4%
016	Actinobacteria	70.04	76.77	34.14	9.15	5.1	1.8%	1.8%	0.5%	0.3%	0.2%
017	Actinobacteria	51.28	30.56	4.35	3.6	13.17	1.3%	0.7%	0.1%	0.1%	0.5%
018	Acidobacteria	47.93	40.72	1.93	2.21	5.86	1.2%	1.0%	0.0%	0.1%	0.2%
019	Actinobacteria	45.91	31.84	151.47	14.36	7.91	1.2%	0.8%	2.3%	0.5%	0.3%
020	(Eukaryote)	42.85	63.14	9.79	1.95	3.26	1.1%	1.5%	0.2%	0.1%	0.1%
021	Proteobacteria	41.37	38.85	22.28	3.01	5.83	1.0%	0.9%	0.3%	0.1%	0.2%
022	Actinobacteria	37.79	40.52	3.09	3.06	8.44	1.0%	1.0%	0.0%	0.1%	0.3%
023	Proteobacteria	30.62	23.84	14.14	3.76	2.94	0.8%	0.6%	0.2%	0.1%	0.1%
024	Planctomycetes	29.87	23.99	2.15	1.52	4.76	0.8%	0.6%	0.0%	0.0%	0.2%

025	Proteobacteria	29.79	39.51	1.04	1.21	1.56	0.7%	0.9%	0.0%	0.0%	0.1%
026	Proteobacteria	29.44	19.38	0.86	1.03	1.71	0.7%	0.5%	0.0%	0.0%	0.1%
027	Chloroflexi	25.7	16.92	2.23	2.05	5.52	0.6%	0.4%	0.0%	0.1%	0.2%
028	Proteobacteria	25.25	43.61	0.46	0.45	1.15	0.6%	1.0%	0.0%	0.0%	0.0%
029	Actinobacteria	24.04	35.61	2.27	1.59	5.83	0.6%	0.9%	0.0%	0.1%	0.2%
030	Actinobacteria	23.89	16.77	1.44	1.18	4.12	0.6%	0.4%	0.0%	0.0%	0.2%
031	Chloroflexi	23.33	22.6	2.36	1.92	6.78	0.6%	0.5%	0.0%	0.1%	0.3%
032	Deinococcus-Thermus	21.41	16.32	0.83	0.76	1.55	0.5%	0.4%	0.0%	0.0%	0.1%
033	Chloroflexi	20.72	16.08	1.32	1.23	3.1	0.5%	0.4%	0.0%	0.0%	0.1%
034	Planctomycetes	18.87	24.04	1.27	1.36	2.89	0.5%	0.6%	0.0%	0.0%	0.1%
035	Acidobacteria	18.2	18.1	0.4	0.35	0.86	0.5%	0.4%	0.0%	0.0%	0.0%
036	Actinobacteria	16.12	3.7	1.68	1.46	4.8	0.4%	0.1%	0.0%	0.0%	0.2%
037	Proteobacteria	15.22	22.88	0.49	0.55	1.03	0.4%	0.5%	0.0%	0.0%	0.0%
038	Actinobacteria	14.83	7.11	0.59	0.75	1.92	0.4%	0.2%	0.0%	0.0%	0.1%
039	Actinobacteria	14.49	12.66	0.35	0.27	0.4	0.4%	0.3%	0.0%	0.0%	0.0%
040	Verrucomicrobia	14.35	32.46	0.24	0.33	0.6	0.4%	0.8%	0.0%	0.0%	0.0%
041	Actinobacteria	13.74	10.95	34.93	4.62	2.42	0.3%	0.3%	0.5%	0.1%	0.1%
042	Actinobacteria	12.54	5.63	0.53	0.67	1.4	0.3%	0.1%	0.0%	0.0%	0.1%
043	Actinobacteria	12.1	9.56	0.84	0.78	1.92	0.3%	0.2%	0.0%	0.0%	0.1%
044	Chloroflexi	11.84	40.72	1.33	0.76	1.21	0.3%	1.0%	0.0%	0.0%	0.0%
045	Firmicutes	10.9	9.01	0.58	0.59	1.39	0.3%	0.2%	0.0%	0.0%	0.1%
046	Proteobacteria	10.73	11.11	0.54	0.53	1.13	0.3%	0.3%	0.0%	0.0%	0.0%
047	Firmicutes	10.61	9.07	7.93	1.02	1.53	0.3%	0.2%	0.1%	0.0%	0.1%
048	Actinobacteria	10.55	6.89	0.66	0.56	1.24	0.3%	0.2%	0.0%	0.0%	0.0%
049	Proteobacteria	9.03	8.71	189.65	18.74	1.34	0.2%	0.2%	2.9%	0.6%	0.1%
050	Actinobacteria	8.87	1.33	0.2	0.22	0.59	0.2%	0.0%	0.0%	0.0%	0.0%
051	Proteobacteria	8.85	12.77	0.41	0.41	0.97	0.2%	0.3%	0.0%	0.0%	0.0%
052	Proteobacteria	8.2	9.4	0.46	0.47	1.12	0.2%	0.2%	0.0%	0.0%	0.0%

053	Proteobacteria	8.15	15.22	19.66	1.29	0.63	0.2%	0.4%	0.3%	0.0%	0.0%
054	Actinobacteria	8.02	16.7	0.61	0.51	0.49	0.2%	0.4%	0.0%	0.0%	0.0%
055	Gemmatimonadetes	7.46	10.24	0.07	0.07	0.12	0.2%	0.2%	0.0%	0.0%	0.0%
056	Proteobacteria	7.3	7.35	0.81	0.37	0.8	0.2%	0.2%	0.0%	0.0%	0.0%
057	Gemmatimonadetes	7.29	10.21	0.08	0.07	0.12	0.2%	0.2%	0.0%	0.0%	0.0%
058	Proteobacteria	7.09	5.43	0.28	0.28	0.39	0.2%	0.1%	0.0%	0.0%	0.0%
059	Proteobacteria	7.05	9.63	0.74	0.46	1.11	0.2%	0.2%	0.0%	0.0%	0.0%
060	Actinobacteria	6.95	15.73	0.43	0.36	1.1	0.2%	0.4%	0.0%	0.0%	0.0%
061	Proteobacteria	6.78	10.29	12.84	2.41	0.78	0.2%	0.2%	0.2%	0.1%	0.0%
062	Proteobacteria	6.67	7.81	12.32	4.62	1.09	0.2%	0.2%	0.2%	0.1%	0.0%
063	Actinobacteria	6.47	4.35	0.8	0.58	1.38	0.2%	0.1%	0.0%	0.0%	0.1%
064	Planctomycetes	6.31	14.27	0.32	0.34	0.74	0.2%	0.3%	0.0%	0.0%	0.0%
065	Proteobacteria	6.17	8.68	0.25	0.21	0.44	0.2%	0.2%	0.0%	0.0%	0.0%
066	Proteobacteria	5.71	4.89	4.45	0.91	0.28	0.1%	0.1%	0.1%	0.0%	0.0%
067	Actinobacteria	5.41	21.28	0.51	0.48	1.27	0.1%	0.5%	0.0%	0.0%	0.1%
068	Actinobacteria	5.38	6.82	0.6	0.46	1.16	0.1%	0.2%	0.0%	0.0%	0.0%
069	Actinobacteria	5.31	4.63	2.84	0.79	0.68	0.1%	0.1%	0.0%	0.0%	0.0%
070	Gemmatimonadetes	5.26	4.87	0.16	0.17	0.35	0.1%	0.1%	0.0%	0.0%	0.0%
071	Gemmatimonadetes	5.26	24.08	0.08	0.09	0.16	0.1%	0.6%	0.0%	0.0%	0.0%
072	Firmicutes	4.79	3.03	174.19	116.16	2.98	0.1%	0.1%	2.7%	3.7%	0.1%
073	Proteobacteria	4.51	1.58	19.67	8.81	3.3	0.1%	0.0%	0.3%	0.3%	0.1%
074	Actinobacteria	4.03	3.01	0.22	0.08	0.14	0.1%	0.1%	0.0%	0.0%	0.0%
075	Planctomycetes	3.92	4.31	0.15	0.13	0.32	0.1%	0.1%	0.0%	0.0%	0.0%
076	Proteobacteria	3.83	5.77	0.23	0.18	0.38	0.1%	0.1%	0.0%	0.0%	0.0%
077	Actinobacteria	3.61	1.07	0.14	0.13	0.27	0.1%	0.0%	0.0%	0.0%	0.0%
078	Firmicutes	3.57	4.66	0.08	0.08	0.16	0.1%	0.1%	0.0%	0.0%	0.0%
079	Actinobacteria	3.53	2.33	0.29	0.21	0.68	0.1%	0.1%	0.0%	0.0%	0.0%
080	Firmicutes	3.46	2.42	165.85	95.91	1.3	0.1%	0.1%	2.6%	3.1%	0.1%

081	Chloroflexi	3.38	3.1	0.45	0.21	0.51	0.1%	0.1%	0.0%	0.0%	0.0%
082	Verrucomicrobia	3.29	5.3	0.57	0.24	0.39	0.1%	0.1%	0.0%	0.0%	0.0%
083	Gemmatimonadetes	3.21	2.83	0.1	0.11	0.29	0.1%	0.1%	0.0%	0.0%	0.0%
084	Actinobacteria	3.17	2.9	5.89	0.62	0.65	0.1%	0.1%	0.1%	0.0%	0.0%
085	Actinobacteria	3.13	2.06	0.28	0.2	0.62	0.1%	0.0%	0.0%	0.0%	0.0%
086	Proteobacteria	3.13	3.18	0.86	0.29	0.41	0.1%	0.1%	0.0%	0.0%	0.0%
087	(Eukaryote)	3.13	3.81	0.25	0.16	0.32	0.1%	0.1%	0.0%	0.0%	0.0%
088	Actinobacteria	2.78	3.01	0.2	0.07	0.12	0.1%	0.1%	0.0%	0.0%	0.0%
089	Firmicutes	2.75	1.18	55.77	11.19	1.64	0.1%	0.0%	0.9%	0.4%	0.1%
090	Actinobacteria	2.73	3.27	0.22	0.2	0.6	0.1%	0.1%	0.0%	0.0%	0.0%
091	Firmicutes	2.73	3.22	0.12	0.1	0.31	0.1%	0.1%	0.0%	0.0%	0.0%
092	Firmicutes	2.7	3.12	0.15	0.12	0.29	0.1%	0.1%	0.0%	0.0%	0.0%
093	Firmicutes	2.57	2.55	0.06	0.07	0.21	0.1%	0.1%	0.0%	0.0%	0.0%
094	Proteobacteria	2.45	2.84	0.21	0.12	0.27	0.1%	0.1%	0.0%	0.0%	0.0%
095	Proteobacteria	2.37	3.66	0.06	0.07	0.18	0.1%	0.1%	0.0%	0.0%	0.0%
096	Actinobacteria	2.24	5.14	0.18	0.16	0.42	0.1%	0.1%	0.0%	0.0%	0.0%
097	Proteobacteria	2.23	2.69	1.81	0.16	0.26	0.1%	0.1%	0.0%	0.0%	0.0%
098	Firmicutes	2.23	3.02	0.07	0.07	0.21	0.1%	0.1%	0.0%	0.0%	0.0%
099	Firmicutes	2.21	3.04	0.06	0.07	0.19	0.1%	0.1%	0.0%	0.0%	0.0%
100	Firmicutes	2.12	1	126.58	241.2	165.95	0.1%	0.0%	2.0%	7.7%	6.6%
101	Firmicutes	2.12	0.82	5.4	4.01	2.75	0.1%	0.0%	0.1%	0.1%	0.1%
102	(Eukaryote)	2.04	2.96	0.06	0.03	0.11	0.1%	0.1%	0.0%	0.0%	0.0%
103	Chloroflexi	2.04	6.99	0.16	0.19	0.45	0.1%	0.2%	0.0%	0.0%	0.0%
104	Firmicutes	2.03	2.95	0.15	0.12	0.23	0.1%	0.1%	0.0%	0.0%	0.0%
105	Proteobacteria	2.01	5.09	0.11	0.09	0.15	0.1%	0.1%	0.0%	0.0%	0.0%
106	Verrucomicrobia	1.94	3.37	0.16	0.09	0.2	0.0%	0.1%	0.0%	0.0%	0.0%
107	(Eukaryote)	1.92	2.58	0.09	0.06	0.12	0.0%	0.1%	0.0%	0.0%	0.0%
108	(Eukaryote)	1.91	1.84	0.13	0.1	0.33	0.0%	0.0%	0.0%	0.0%	0.0%

109	(Eukaryote)	1.87	1.82	0.16	0.1	0.36	0.0%	0.0%	0.0%	0.0%	0.0%
110	(Eukaryote)	1.86	1.64	0.17	0.11	0.34	0.0%	0.0%	0.0%	0.0%	0.0%
111	Chloroflexi	1.86	2.15	0.19	0.12	0.33	0.0%	0.1%	0.0%	0.0%	0.0%
112	(Eukaryote)	1.84	1.74	0.14	0.11	0.38	0.0%	0.0%	0.0%	0.0%	0.0%
113	(Eukaryote)	1.82	1.73	0.14	0.1	0.35	0.0%	0.0%	0.0%	0.0%	0.0%
114	Firmicutes	1.45	2.17	243.63	178.61	1.37	0.0%	0.1%	3.8%	5.7%	0.1%
115	Planctomycetes	1.35	3.98	0.04	0.04	0.09	0.0%	0.1%	0.0%	0.0%	0.0%
116	Firmicutes	1.27	1.48	46.81	1.04	0.18	0.0%	0.0%	0.7%	0.0%	0.0%
117	Firmicutes	1.27	0.65	5.05	3.73	2.47	0.0%	0.0%	0.1%	0.1%	0.1%
118	Proteobacteria	1.01	1.49	81.14	25.14	0.42	0.0%	0.0%	1.3%	0.8%	0.0%
119	Firmicutes	0.97	0.89	26.61	3.23	0.13	0.0%	0.0%	0.4%	0.1%	0.0%
120	Firmicutes	0.9	0.48	47.86	35.84	5.82	0.0%	0.0%	0.7%	1.2%	0.2%
121	Firmicutes	0.87	0.47	9.4	0.42	0.15	0.0%	0.0%	0.1%	0.0%	0.0%
122	Firmicutes	0.84	0.44	288.17	450.93	386.64	0.0%	0.0%	4.4%	14.5%	15.3%
123	Actinobacteria	0.81	0.5	5.33	2.65	0.24	0.0%	0.0%	0.1%	0.1%	0.0%
124	Actinobacteria	0.78	6.22	0.04	0.05	0.09	0.0%	0.1%	0.0%	0.0%	0.0%
125	Bacteroidetes	0.77	3.68	0.27	0.02	0.03	0.0%	0.1%	0.0%	0.0%	0.0%
126	Firmicutes	0.65	0.48	10.55	25.78	14.72	0.0%	0.0%	0.2%	0.8%	0.6%
127	Firmicutes	0.64	0.69	231.09	544.08	973.49	0.0%	0.0%	3.6%	17.5%	38.6%
128	Firmicutes	0.54	0.41	0.25	0.21	0.36	0.0%	0.0%	0.0%	0.0%	0.0%
129	Firmicutes	0.52	0.39	35.53	4.96	0.11	0.0%	0.0%	0.5%	0.2%	0.0%
130	Firmicutes	0.39	0.21	22.32	53.55	12.23	0.0%	0.0%	0.3%	1.7%	0.5%
131	Firmicutes	0.34	0.2	7.08	1.27	0.11	0.0%	0.0%	0.1%	0.0%	0.0%
132	Firmicutes	0.25	0.38	90.58	143.74	328.41	0.0%	0.0%	1.4%	4.6%	13.0%
133	Firmicutes	0.22	0.2	20.27	29.89	62.32	0.0%	0.0%	0.3%	1.0%	2.5%
134	Firmicutes	0.08	0.07	25.36	45.31	0.09	0.0%	0.0%	0.4%	1.5%	0.0%
135	Firmicutes	0.07	0.04	0.58	15.25	0.06	0.0%	0.0%	0.0%	0.5%	0.0%
136	Firmicutes	0.02	0.02	0.18	31.68	0.11	0.0%	0.0%	0.0%	1.0%	0.0%

137	Firmicutes	0.01	0.29	0.21	0.38	0.22	0.0%	0.0%	0.0%	0.0%	0.0%
138	Firmicutes	0.01	0.01	1.91	0.4	22.57	0.0%	0.0%	0.0%	0.0%	0.9%
