# Supplementary material for: Ionic Liquids Impact the Bioenergy Feedstock-Degrading Microbiome and Transcription of Enzymes Relevant to Polysaccharide Hydrolysis
Source: mSystems. 2016 Dec 13;1(6):e00120-16. doi: 10.1128/mSystems.00120-16 (PMC5155067; doi:10.1128/mSystems.00120-16)
Supplement: Table S4 [file sys006162071st4.pdf]

Table S4. Mapping rates of genomic and transcriptomic sequencing reads against the co-assembled metagenome

	Samples	Mapping Rate
Metagenomic reads	Inoculum	97.06%
	0% IL	97.22%
	0.5% IL	96.71%
	1% IL	96.04%
	2% IL	94.36%
Metatranscriptomic reads	Inoculum	91.72%
	0% IL	92.14%
	0.5% IL	96.95%
	1% IL	97.68%
	2% IL	94.58%
