# Supplementary material for: Ionic Liquids Impact the Bioenergy Feedstock-Degrading Microbiome and Transcription of Enzymes Relevant to Polysaccharide Hydrolysis
Source: mSystems. 2016 Dec 13;1(6):e00120-16. doi: 10.1128/mSystems.00120-16 (PMC5155067; doi:10.1128/mSystems.00120-16)
Supplement: Table S5 [file sys006162071st5.pdf]

Table S5. Shannon diversity, richness and Pielou's evenness values for microbial communities by different treatments.

	Shannon	Richness	Evenness
Inoculum	2.93	117	0.62
0% IL	2.97	190	0.57
0.5% IL	2.71	114	0.57
1% IL	2.17	113	0.46
2% IL	1.74	143	0.35
