# Supplementary material for: Ionic Liquids Impact the Bioenergy Feedstock-Degrading Microbiome and Transcription of Enzymes Relevant to Polysaccharide Hydrolysis
Source: mSystems. 2016 Dec 13;1(6):e00120-16. doi: 10.1128/mSystems.00120-16 (PMC5155067; doi:10.1128/mSystems.00120-16)
Supplement: Table S6 [file sys006162071st6.pdf]

Table S6. Summarized RPKM values for the genome bins.

		Inoculum	0% IL	0.5% IL	1% IL	2% IL
001	Proteobacteria	166782.49	173878.89	7304.68	1512.42	6200.93
002	Proteobacteria	35526.28	30206.95	962.82	640.00	10952.59
003	Proteobacteria	23829.19	17977.87	213559.52	51675.57	34338.97
004	Bacteroidetes	76907.20	66155.43	92230.18	638.76	411.44
005	Chloroflexi	110856.28	153688.24	890.79	535.75	3765.55
006	Proteobacteria	21359.45	35243.91	279113.90	7884.95	3681.29
007	Planctomycetes	8650.10	17172.62	764.77	438.70	7104.08
008	Proteobacteria	39025.24	18011.05	72936.34	3637.03	4552.50
009	Proteobacteria	94722.48	102766.90	1618.39	1202.40	5864.00
010	Actinobacteria	36239.34	53882.00	219238.40	4560.51	6868.54
011	Actinobacteria	18006.55	7921.03	489.04	660.49	7584.30
012	(unclassified)	1141.46	119.60	47.04	193.57	1117.14
013	Gemmatimonadetes	8074.74	9054.15	110.66	94.46	2198.89
014	Actinobacteria	17329.86	13394.83	4357.99	2514.85	6912.75
015	Proteobacteria	65443.70	29284.13	457.52	294.60	2789.44
016	Actinobacteria	27785.96	44570.24	8749.04	1897.19	3982.17
017	Actinobacteria	11090.08	6783.82	76.92	81.13	2879.49
018	Acidobacteria	13423.18	10607.79	102.27	61.31	1440.05
019	Actinobacteria	15371.47	7174.61	60822.81	7299.44	2022.29
020	(Eukaryote)	N/A[a]	N/A	N/A	N/A	N/A
021	Proteobacteria	25373.22	19580.25	23485.31	650.34	2063.98
022	Actinobacteria	682.26	978.03	95.78	93.69	1904.87
023	Proteobacteria	6550.33	5425.00	5441.97	264.98	2236.11
024	Planctomycetes	18487.63	12222.14	144.46	107.09	1280.91
025	Proteobacteria	7407.22	7930.81	81.07	45.55	173.68

026	Proteobacteria	11040.73	6862.36	27.73	26.84	170.82
027	Chloroflexi	5864.19	3685.06	173.98	118.43	2282.14
028	Proteobacteria	30105.24	54050.27	45.68	26.21	754.67
029	Actinobacteria	1033.54	1831.07	50.88	36.95	1396.71
030	Actinobacteria	2374.55	1813.98	74.53	40.91	1294.98
031	Chloroflexi	10706.11	12596.71	149.91	132.94	2647.89
032	Deinococcus-Thermus	1099.10	1222.48	27.03	15.74	387.36
033	Chloroflexi	74427.12	49584.43	82.86	88.81	376.02
034	Planctomycetes	46833.18	54338.30	46.77	55.57	437.59
035	Acidobacteria	33405.37	31320.19	148.36	70.37	440.70
036	Actinobacteria	966.85	206.05	35.63	26.50	778.65
037	Proteobacteria	13867.91	24032.33	40.06	28.02	326.04
038	Actinobacteria	1210.36	338.53	7.21	11.61	101.05
039	Actinobacteria	1465.67	1704.06	86.21	52.86	367.47
040	Verrucomicrobia	3742.84	6442.78	14.02	15.88	111.67
041	Actinobacteria	5756.26	5375.91	55617.00	3322.30	1956.22
042	Actinobacteria	548.94	214.64	13.11	26.29	51.66
043	Actinobacteria	893.81	865.12	33.82	21.08	97.09
044	Chloroflexi	29304.86	73324.08	56.32	66.57	177.34
045	Firmicutes	2467.29	2354.14	48.32	24.34	339.97
046	Proteobacteria	1480.36	1671.65	33.06	16.91	476.46
047	Firmicutes	7251.90	8041.24	7573.66	436.91	142.46
048	Actinobacteria	1047.49	883.62	53.01	22.89	456.84
049	Proteobacteria	1262.59	1310.89	73647.40	68773.99	398.29
050	Actinobacteria	1599.39	378.10	33.13	14.69	341.94
051	Proteobacteria	10624.28	12678.36	23.46	26.84	123.44
052	Proteobacteria	2274.04	2175.75	36.62	27.22	565.75

053	Proteobacteria	966.08	2000.58	4039.70	57.30	127.02
054	Actinobacteria	3608.75	10355.82	107.15	38.66	259.46
055	Gemmatimonadetes	162.13	160.00	0.71	0.00	8.91
056	Proteobacteria	6037.23	6430.12	418.00	45.17	216.94
057	Gemmatimonadetes	33.34	58.68	0.52	0.00	6.11
058	Proteobacteria	1726.12	1197.57	14.87	7.35	49.43
059	Proteobacteria	867.64	996.95	642.26	3791.82	1666.33
060	Actinobacteria	521.20	1214.42	15.60	4.18	294.27
061	Proteobacteria	5990.29	5664.13	18949.98	3425.77	208.64
062	Proteobacteria	1969.44	1563.88	4431.12	12096.72	142.65
063	Actinobacteria	108.74	89.92	25.35	15.89	246.44
064	Planctomycetes	1745.67	3658.48	8.95	12.24	83.80
065	Proteobacteria	647.64	1305.02	21.37	17.38	183.10
066	Proteobacteria	1986.74	1736.30	2469.75	49.13	69.27
067	Actinobacteria	184.45	796.74	8.98	11.95	220.77
068	Actinobacteria	340.45	436.85	14.00	12.24	243.50
069	Actinobacteria	483.68	306.42	190.22	28.04	27.37
070	Gemmatimonadetes	5820.44	5083.48	30.23	9.04	122.61
071	Gemmatimonadetes	496.84	2899.16	10.05	3.93	57.91
072	Firmicutes	417.85	380.54	57336.82	100632.33	321.46
073	Proteobacteria	3776.61	628.47	3705.03	650.49	1060.96
074	Actinobacteria	772.47	660.00	27.60	7.96	108.07
075	Planctomycetes	1806.41	2281.63	50.80	16.85	195.71
076	Proteobacteria	1394.28	2298.46	22.64	9.74	135.25
077	Actinobacteria	725.90	361.12	44.61	13.91	241.96
078	Firmicutes	3518.54	5746.19	17.72	7.09	65.59
079	Actinobacteria	346.18	227.77	10.19	6.47	191.36
080	Firmicutes	294.71	200.65	29724.17	27648.43	213.55

081	Chloroflexi	4093.47	4439.99	514.01	130.08	928.08
082	Verrucomicrobia	676.73	878.30	40.87	27.21	64.39
083	Gemmatimonadetes	1193.99	1117.05	8.80	4.46	130.31
084	Actinobacteria	4651.87	4038.40	9954.43	88.21	494.25
085	Actinobacteria	112.72	83.55	6.38	3.48	132.84
086	Proteobacteria	4832.67	4551.80	1214.56	179.61	435.05
087	(Eukaryote)	N/A	N/A	N/A	N/A	N/A
088	Actinobacteria	387.21	569.04	24.14	3.29	57.77
089	Firmicutes	2723.90	335.04	5226.44	1778.56	332.91
090	Actinobacteria	149.09	211.61	9.84	6.36	188.86
091	Firmicutes	225.45	286.09	4.71	0.97	16.49
092	Firmicutes	461.53	707.20	7.63	4.99	26.77
093	Firmicutes	881.46	615.33	3.95	1.63	20.51
094	Proteobacteria	2340.81	3367.80	104.61	59.10	247.13
095	Proteobacteria	865.29	1049.10	5.76	2.73	72.52
096	Actinobacteria	597.68	1957.77	9.97	5.82	113.69
097	Proteobacteria	457.52	554.12	289.97	4.12	37.49
098	Firmicutes	121.16	203.21	1.22	0.72	20.97
099	Firmicutes	411.26	332.71	2.80	0.95	13.76
100	Firmicutes	51.87	44.09	1958.33	102096.85	140205.43
101	Firmicutes	289.55	249.58	579.60	1970.10	7307.44
102	(Eukaryote)	N/A	N/A	N/A	N/A	N/A
103	Chloroflexi	415.34	1172.91	9.17	9.32	123.29
104	Firmicutes	517.21	912.28	6.60	1.78	12.72
105	Proteobacteria	728.87	1219.16	11.77	2.59	39.41
106	Verrucomicrobia	2626.11	6674.49	252.30	13.33	54.61
107	(Eukaryote)	N/A	N/A	N/A	N/A	N/A
108	(Eukaryote)	N/A	N/A	N/A	N/A	N/A

109	(Eukaryote)	N/A	N/A	N/A	N/A	N/A
110	(Eukaryote)	N/A	N/A	N/A	N/A	N/A
111	Chloroflexi	850.13	964.96	26.51	14.70	217.55
112	(Eukaryote)	N/A	N/A	N/A	N/A	N/A
113	(Eukaryote)	N/A	N/A	N/A	N/A	N/A
114	Firmicutes	263.56	470.89	61497.94	177435.29	164.16
115	Planctomycetes	1090.24	3147.50	5.99	0.00	20.47
116	Firmicutes	166.93	403.41	21565.53	282.15	36.28
117	Firmicutes	419.34	364.44	250.62	2269.72	11035.83
118	Proteobacteria	552.57	453.03	8359.32	2023.89	170.20
119	Firmicutes	348.70	398.68	2563.11	625.27	21.04
120	Firmicutes	15.52	12.96	1533.52	2120.01	1357.08
121	Firmicutes	152.90	103.60	4105.48	20.74	39.63
122	Firmicutes	25.45	74.16	9727.14	52760.33	34363.81
123	Actinobacteria	326.56	182.97	7763.75	7525.30	301.32
124	Actinobacteria	162.37	2746.39	4.89	0.93	23.00
125	Bacteroidetes	1759.71	5152.19	425.38	6.63	6.00
126	Firmicutes	21.77	22.93	330.62	2623.72	985.69
127	Firmicutes	347.59	220.53	8837.61	146184.44	519513.72
128	Firmicutes	26.87	43.23	6.66	39.96	8.20
129	Firmicutes	93.72	121.70	9749.02	1612.44	13.41
130	Firmicutes	19.12	5.55	499.39	50964.11	611.31
131	Firmicutes	36.91	37.74	2248.17	74.00	4.61
132	Firmicutes	6.79	15.02	2624.72	211736.00	299817.60
133	Firmicutes	24.38	15.83	579.29	31954.52	33359.04
134	Firmicutes	6.85	6.28	4444.75	71975.65	17.90
135	Firmicutes	17.18	4.83	70.05	5556.16	8.22
136	Firmicutes	0.00	0.00	7.78	61765.55	31.40

137	Firmicutes	0.85	2.92	7.59	136.12	166.21
138	Firmicutes	0.00	0.00	14.44	7.26	796.74
Unbinned [b]		662413.97	714711.19	609576.20	1295559.72	1369481

[a] The RPKM values of eukaryotic bins are ignored since MaxBin was not designed to bin eukaryotes, and the RPKM estimation of eukaryotic genes may be biased using our metatranscriptomics analysis pipeline due to the exon-intron structure of eukaryotic organisms.

[b] The RPKM values of unbinned genes are summarized here. The summed RPKM values may include unbinned eukaryotic genes and may be over-estimated.
