# Supplementary material for: Ionic Liquids Impact the Bioenergy Feedstock-Degrading Microbiome and Transcription of Enzymes Relevant to Polysaccharide Hydrolysis
Source: mSystems. 2016 Dec 13;1(6):e00120-16. doi: 10.1128/mSystems.00120-16 (PMC5155067; doi:10.1128/mSystems.00120-16)
Supplement: Table S7 [file sys006162071st7.pdf]

Table S7. Top 5 expressed genes in Bin 127 for all five samples.

Gene	Gene product	RPKM				
		Inoculum	0% IL	0.5% IL	1% IL	2% IL
scaffold_119436_3	hypothetical protein	342.7457	216.213	2420.401	13730.02	132053.3
scaffold_172955_4	Membrane protein TerC, possibly involved in tellurium resistance	1.594824	1.611396	451.1241	16632.19	72931.25
scaffold_42143_5	Arabinose efflux permease	0	0	176.8868	9105.197	43112.1
scaffold_218866_2	Isocitrate lyase	0	0	1029.381	20086.59	36461.34
scaffold_38470_1	hypothetical protein (ABC transporter permease)	0	0	526.4152	8014.849	31638.67
