# Supplementary material for: Ionic Liquids Impact the Bioenergy Feedstock-Degrading Microbiome and Transcription of Enzymes Relevant to Polysaccharide Hydrolysis
Source: mSystems. 2016 Dec 13;1(6):e00120-16. doi: 10.1128/mSystems.00120-16 (PMC5155067; doi:10.1128/mSystems.00120-16)
Supplement: Table S8 [file sys006162071st8.pdf]

Table S8. Percentages of KO annotations matched to EC numbers

KEGG annotation	Enzyme class	Corresponding EC number	Matching accuracy[1]
K00925	Acetate kinase	EC 2.7.2.1	100%
K01179, K19356, K19357 and K20542	Endoglucanase	EC 3.2.1.4	75.61%
K01225, K19668	Cellobiohydrolase	EC 3.2.1.91	14.29%
K01188, K05349, K05350	Beta-glucosidase	EC 3.2.1.21	93.28%
K01181, K13465	Xylanase	EC 3.2.1.8	96.84%

[1] Percentage of KEGG annotation matched to corresponding EC number
